# Multi-paradigm Vision Transformer ensemble with regional attention and MLP meta-fusion for explainable dermoscopic skin lesion classification

**DOI:** 10.3389/fmed.2026.1864593

**Published:** 2026-08-13

**Authors:** Nemili Sravani, Srinivas Koppu

**Affiliations:** School of Computer Science Engineering and Information Systems, Vellore Institute of Technology, Vellore, India

**Keywords:** DeiT, explainable artificial intelligence, MLP meta-learner, Regional Attention Wrapper, skin lesion classification, Swin Transformer, Vision Transformer

## Abstract

**Introduction:**

The automated classification of dermoscopic skin lesions is inherently challenging due to pronounced class imbalance, minimal inter-class variance, visual similarity across lesion types, and the requirement for clinically interpretable predictive outcomes.

**Methods:**

The present study designed a heterogeneous Vision Transformer ensemble framework for seven-class skin lesion classification using the HAM10000 dataset. The framework integrates three architecturally distinct backbones Swin-Tiny, ViT-Base, and DeiT-Small enhanced with a novel Regional Attention Wrapper (RAW) for spatially selective feature aggregation. The generated outputs are combined via a stacking protocol wherein a trained MLP meta-learner resolves class-aware disagreements among the models. Class imbalance is addressed using class-adaptive augmentation with class-weighted focal loss and MixUp regularisation.

**Results:**

The proposed framework achieved 98.37% accuracy, weighted F1-score of 98.39%, and mean AUC of 0.999, surpassing all three individual backbones across all metrics. MEL misclassifications were reduced by 78% compared to the weakest baseline, confirmed by McNemar's test (*p* < 0.0001). Comprehensive ablation studies validate the contributions of the RAW module, each ensemble component, and each augmentation strategy. Three-fold cross-validation yields a mean accuracy of 96.14 ± 0.36% and bootstrap confidence interval analysis confirms the reliability and reproducibility of the reported results.

**Discussion:**

An exhaustive explainability framework comprising Regional Attention Maps, GradCAM++, SHAP, and t-SNE provides complementary spatial, gradient-based, pixel-level, and embedding-level interpretability, ensuring clinical trust, transparency, and trustworthiness expected from an automated dermoscopy system.

## 1 Introduction

Skin cancer is considered one of the most prevalent and rapidly rising malignant diseases worldwide, with melanoma representing its deadliest form (1). Early detection and accurate diagnosis of melanoma can lead to a 5-year survival rate exceeding 98%; however, if detected at an advanced stage, the survival rate falls below 25%. Dermoscopy is a non-invasive imaging technique widely used in skin cancer detection that relies on magnification of the subsurface skin structure (2). However, accurate diagnosis of the disease requires dermoscopic interpretation based on substantial expertise, which is often subject to inter-observer variability. These challenges are particularly prominent in cases of visually ambiguous lesions, namely melanoma vs. melanocytic nevi. The use of computer-aided diagnosis tools using deep learning techniques enables consistent, scalable, and objective lesion classification, mitigating these challenges (3). The use of Convolutional Neural Networks (CNNs), including ResNet, DenseNet, and EfficientNet, has yielded promising results on dermoscopic benchmarks such as the HAM10000 dataset. The ensemble framework has further enhanced accuracy by leveraging complementary features across the models (4). Nevertheless, the performance of CNN methods remains constrained by localized receptive fields, limiting their ability to model long-range spatial dependencies crucial for detecting significant attributes such as lesion asymmetry, border irregularity, and global color patterns.

Vision Transformers (ViTs) have emerged as an efficient alternative to CNNs for image classification, leveraging global self-attention mechanisms to capture long-range relationships from the first layer (5). In this context, variants such as Swin Transformers, which use hierarchical shifted-window attention, and DeiT, which employs knowledge distillation, have also contributed to the computational efficiency and performance of the ViT framework. The application of ViT-based frameworks in dermoscopic analysis has thus gained immense momentum due to their promising results, generating superior outcomes in contrast to traditional CNNs, considering significant benchmarks (6). But the majority of existing ViT-based approaches for skin lesion classification rely on a single backbone architecture, limiting the diversity of learned feature representations and further reducing the robustness of the model in generating predictive outcomes (7). On the contrary, Ensemble methods integrate multiple ViT backbones, providing a natural approach to achieving enhanced performance. However, existing ViT-based ensemble approaches in medical imaging employ homogeneous architectures that combine multiple instances of the same architecture, thereby constraining error distributions and the complementarity of the framework (8). Notably, conventional fusion models such as soft voting and naive averaging treat all model outputs equally, neglecting to leverage inter-model complementarity in settings where diverse architectures are combined. The third limitation concerns feature aggregation, wherein standard pooling techniques such as CLS token extraction and global pooling treat all spatial regions uniformly, overlooking that dermoscopic lesions are spatially localized to specific areas rather than distributed across the entire image due to discriminative features. One of the major challenges in the clinical deployment of deep learning models in real-time is their lack of explainability (9). Regulatory approvals by established healthcare administrative bodies and clinical adoption of such automated diagnosis systems rely on the basic need for the models' decisions to be interpretable and traceable to specific image regions (10). Although various explainability models, namely GradCAM, SHAP, and attention visualization, have been used for skin lesion classification, comprehensive multi-method explainable frameworks that can validate models' decisions from diverse perspectives remain largely unexplored. To address this research gap, this study introduces a heterogeneous Vision Transformer ensemble framework for dermoscopic skin lesion classification, using the HAM10000 dataset. The unique novel contributions of the proposed framework include:

1. A heterogeneous ensemble comprising three architecturally diverse Vision Transformer backbones, Swin-Tiny, ViT-Base, and DeiT-Small, is selected to maximize complementary inductive biases encompassing local window attention, global self-attention, and distillation-based training paradigms.
2. A Regional Attention Wrapper, a unified learnable spatial attention module, is applied consistently across all three backbones, ensuring spatially selective feature aggregation that emphasizes diagnostically significant lesion regions, thereby suppressing uninformative background.
3. An MLP Meta-Learner is trained using a stacking protocol on the concatenated softmax probability outputs of all three backbones, enabling non-linear, class-aware resolution of inter-model disagreements.
4. A multi-method explainability framework involving four complementary XAI methods Regional Attention Map visualization, Ensemble GradCAM++, SHAP GradientExplainer, and t-SNE feature space analysis ensures spatial, gradient-based, pixel-level, and embedding-level interpretability of the predictive outcomes of the model.
5. Statistical validation through McNemar's test with Yates' continuity correction performed across all six pairwise model comparisons, establishing that the proposed ensemble statistically significantly surpasses the performance of each backbone (all *p* < 0.0001).
6. Comprehensive experimental validation through RAW module ablation study, ensemble component ablation study, data augmentation ablation study, three-fold stratified cross validation achieving mean accuracy of 96.14 ± 0.36% and bootstrap confidence interval analysis confirming 95% confidence intervals of (97.55%, 99.09%) for accuracy, (97.60%, 99.09%) for F1-score, and (0.9978, 0.9998) for AUC, collectively establishing reliability and reproducibility of proposed model.

The proposed framework achieves an overall classification accuracy of 98.37%, a weighted F1-score of 98.39%, and a mean AUC of 0.999 on the HAM10000 validation set, outperforming all three individual backbone models and establishing a new state-of-the-art on this benchmark. The rest of the study is organized as follows: Section 2 covers related work; Section 3 describes the proposed methodology; Section 4 presents experimental results; and Section 5 concludes the study.

## 2 Related works

Automated skin lesion classification has advanced rapidly with AI and deep learning models, evolving from basic CNNs to transfer learning, ensemble methods, transformer architectures, and explainable AI systems. In Raghavendra et al. (11), the authors proposed a deep CNN model with global average pooling for classifying skin lesions. The main goal was to build a lightweight model that can detect skin cancer early with optimal accuracy. The model was tested on the HAM10000 dataset using black hat filtering to remove hair artifacts and data augmentation to handle class imbalance. The model achieved 97.20% accuracy with 1,08,471 parameters, which outperformed VGG16, ResNet50, MobileNetV2, and DenseNet121. Future work can be expanded by adding attention mechanisms and transformer-based architectures to improve model interpretability. Another interesting work in Cheng et al. (12) proposed MobileNet-MFS, which is an improved version of MobileNet V3 that replaces the attention block with a new Fused Spatial-Channel Attention mechanism. This model helps capture both spatial and channel information simultaneously. The experimental findings were carried out on the ISIC 2019 dataset. The results in this study showed precision, recall, and F1-score of 0.8732, 0.8751, and 0.8730, respectively. In the future, the work can be expanded by incorporating better explainability tools to enhance trustworthiness in healthcare applications. Interestingly, the recent work in Govindu et al. (13) addressed training instability and class imbalance by alternately combining Batch Normalization and Group Normalization layers within the same CNN. During this process, the authors also used a three-step data balancing strategy involving SMOTE, Tomek links undersampling, and class-weighted oversampling. The model was trained on the HAM10000 dataset. The analytical results show that the proposed model achieved 96.4% accuracy and improved F1-scores, especially for minority classes such as melanoma and dermatofibroma, by 12%–15%. The results are confirmed by McNemar's test with *p* < 0.01. Multi-center validation using diverse demographic data is recommended for future work. Another application of deep learning for skin disease classification and segmentation can be found in Jaber and Akbas (14), where the authors combined a deep learning model with the Binary Harris Hawks Optimization algorithm to improve melanoma segmentation and classification. During this process, the optimization algorithm finds the optimal threshold for binarizing skin lesion images, while GoogleNet and ResNet-50 handle feature extraction and classification. The proposed method was trained on the ISIC 2016, ISIC 2017, and ISIC 2019 datasets and achieved accuracies of 96.35%, 98.44%, and 97.01%, outperforming SVM, Decision Tree, and K-NN classifiers. Future work can be extended to include diverse skin types and expert-annotated images to improve generalizability across real-world healthcare applications.

While these CNN-based models achieved better performance, they still struggle to capture long-range spatial relationships and handle severe class imbalance, prompting researchers to explore transfer learning and ensemble-based methods. Motivated by such challenges, the authors in Shakya et al. (15) systematically compared three strategies for skin lesion classification using pre-trained networks, namely VGG19, ResNet-18, and MobileNet_V2. The strategies included using them as fine-tuned classifiers, as feature extractors with traditional ML classifiers such as SVM, DT, Naive Bayes, and KNN, and as combined feature extractors in a hybrid model. The proposed model was evaluated on the ISIC 2018 dataset, which contains 3,300 images. The results of the study show that the proposed model achieved the highest accuracy of 92.87% by combining MobileNet_V2 and ResNet-18 features with an SVM classifier. The authors have highlighted the need for multi-class extension and transformer-based representations in future work. Another similar work in Saeed et al. (16) proposed a multimodal ensemble framework that combines five CNN backbone models: ResNet50, Xception, MobileNet, EfficientNetB0, and DenseNet121. During this process, the authors used an adaptive weighted ensemble strategy that learns each model's contribution during the training phase. The image features are combined with patient metadata such as age, sex, and anatomical site. Class imbalance is handled using SMOTE. The results were carried out on the ISIC 2018 and ISIC 2019 datasets. The results discovered in the study show that the proposed model achieved 93.2% accuracy and 97.3% AUC on ISIC 2018, and 91.1% accuracy and 95.5% AUC on ISIC 2019. Grad-CAM was used for clinical interpretability. Future work can be extended by showing how metadata fusion helps in individual predictions accurately and achieves better generalization across diverse clinical datasets.

Ensemble methods improve accuracy by combining features from multiple models. However, there are some limitations, such as reliance on conventional CNN backbones, dependence on metadata, and a lack of learned mechanisms to resolve class-specific disagreements between models for confusing minority lesion classes. This gap motivated the use of transformer-based architectures, enabling more effective feature modeling and higher-level decision-making. The work in Ozdemir and Pacal (17) proposed a hybrid model that combines ConvNeXtV2 blocks in the first two stages for local feature extraction and separable self-attention layers in the last two stages for global context modeling. The model was trained on the ISIC 2019 dataset, which the authors augmented and used for transfer learning. An accuracy of 93.48%, 93.24% precision, 90.70% recall, and 91.82% F1-score were achieved, outperforming more than ten CNN models and ten ViT models using 21.92 million parameters. The authors acknowledged that various explainability methods, such as attention heatmaps, saliency maps, and ensemble meta-learning, are required to further improve clinical trust. Another interesting research in Najjar et al. (18) presents VGG19-RSPDA-ViT, which combines VGG19 for local feature extraction with a Vision Transformer for global context modeling. A key novelty of this work is the Rotated and Shifted Patch Data Augmentation strategy. During this process, sixteen different rotated and shifted versions of each feature map are generated before tokenization. This technique helps the model become more robust to orientation changes. The experimentation was carried out on MSK10000, HAM10000, and PH2 datasets, attaining the accuracies of 97.9%, 97.1%, and 98.67%. Grad-CAM was used to confirm that the model focused on lesion regions. Class-aware focal loss, heterogeneous ensemble strategies, and advanced explainability techniques such as SHAP and transformer attention maps could be considered for future work. A lightweight model, SkinSwinViT, is introduced in Tang et al. (19) and is based on the Swin Transformer. The proposed model uses a local-global hierarchical attention mechanism to capture both fine local patterns and broader global context from dermoscopic images. The model was evaluated on the ISIC 2018 dataset, achieving 97.88% accuracy, 97.79% F1-score, and 99.36% specificity, outperforming the standard Swin Transformer, ViT, and ResNet50 baselines. Exploring cross-modal fusion and stronger interpretability methods is lacking and is needed to improve clinical acceptance. The work in Li et al. (20) introduces the Feature Enhancement and Interaction Transformer module (FeIT). It is designed like a plug-and-play component that can be added to any existing CNN backbone model without changing pretrained weights. The proposed model captures all global feature correlations and inter-sample distribution information to improve classification under class imbalance. The model was tested on the white patchy skin lesion dataset, which contains 1,557 images across four classes; all images were collected from Xijing Hospital. DenseNet121 with the FeiT module achieved 92.65% accuracy, 92.74% F1-score, and 0.98 AUC. The experimental findings demonstrate outstanding performance compared to standalone CNN baselines. However, we observed that applying the module to earlier backbone stages improves the capture of comprehensive global features, and that using XAI can enhance model transparency. Recent works have also demonstrated the effectiveness of ensemble frameworks with integrated explainability in broader medical image classification tasks. This work highlights the importance of quantitatively evaluating explainability in medical image classification. It reinforces the clinical relevance of the multi-method XAI framework employed in the present study for dermoscopic skin lesion classification.

Table 1 consolidates all reviewed methods, summarizing their architectures, datasets, key metrics, explainability approaches, and the identified limitations that motivated the proposed framework. The reviewed works reveal several research gaps that the present study addresses. CNN-based models (11–14) are efficient, but they cannot capture the long-range spatial dependencies required for accurate seven-class dermoscopic classification. Second, ensemble approaches (15, 16) rely on conventional CNN backbones, depend on metadata, and lack a learned mechanism to resolve class-specific disagreements between models for confusing minority lesion classes. Third, transformer-based models (17–20) use single-backbone models that are prone to class-aware errors. A meta-learner to combine multiple models intelligently is not included. Fourth, explainability across all reviewed works is very limited and relies solely on single-method saliency maps. These cannot provide the full spatial, gradient-based, pixel-level, and embedding-level insights even though they are very much needed for clinical trust. To address these limitations, this study proposes a heterogeneous Vision Transformer ensemble that integrates Swin-Tiny, ViT-Base, and DeiT-Small. The model is enhanced by a Regional Attention Wrapper, an MLP meta-learner for class-aware fusion, and class-adaptive augmentation with focal loss and MixUp. XAI approaches like Regional Attention Maps, GradCAM++, SHAP, and t-SNE are used to ensure interpretability and clinical trustworthiness in an automated dermoscopy system.

**Table 1:** Summary of reviewed methods for dermoscopic skin lesion classification.

References	Method	Architecture	Dataset	Key metrics	XAI	Limitation
Raghavendra et al. (11)	Deep CNN+GAP	Lightweight CNN	HAM10000	Acc: 97.20%	None	No attention, no XAI
Cheng et al. (12)	MobileNet-MFS	MobileNetV3 + FSCA	ISIC 2019	F1: 0.873	None	Limited explainability
Govindu et al. (13)	BN+GN CNN	Custom CNN	HAM10000	Acc: 96.40%	None	No transformer, no XAI
Jaber et al. (14)	HHO+GoogleNet	GoogleNet + ResNet-50	ISIC 2016/17/19	Acc: 96.35%–98.44%	None	No XAI, limited diversity
Shakya et al. (15)	Transfer+SVM	VGG19 + ResNet-18 + MobileNetV2	ISIC 2018	Acc: 92.87%	None	No multi-class, no transformer
Saeed et al. (16)	Multimodal CNN	ResNet50 + EfficientNetB0 + DenseNet121	ISIC 2018/19	Acc: 93.20%, AUC: 0.973	GradCAM	Metadata dependent, no meta-learner
Ozdemir and Pacal (17)	ConvNeXtV2+ViT	Hybrid CNN-ViT	ISIC 2019	Acc: 93.48%, F1: 0.918	None	Single backbone, no XAI
Najjar et al. (18)	VGG19-RSPDA-ViT	VGG19 + ViT	HAM10000/PH2	Acc: 97.10%–98.67%	GradCAM	No SHAP, no meta-learner
Tang et al. (19)	SkinSwinViT	Swin Transformer	ISIC 2018	Acc: 97.88%, F1: 0.978	None	Single backbone, limited XAI
Li et al. (20)	FeIT+DenseNet	DenseNet121 + FeIT	Xijing Hospital	Acc: 92.65%, AUC: 0.980	None	Small dataset, no XAI
–	**Proposed**	**Swin-Tiny + ViT-Base + DeiT-Small + MLP**	**HAM10000**	**Acc: 98.37%, AUC: 0.999**	**4 methods**	

Bold values indicate the best performance for each metric.

## 3 Methodology

This section presents the complete methodology of the proposed system, organized into several sections: dataset preparation, augmentation, loss formulation, backbone architectures, the Regional Attention Wrapper, the MLP Meta-Learner, the training protocol, and the explainability framework.

### 3.1 Dataset description

The HAM10000 dataset (21, 27) consists of 10,015 dermoscopic images which are grouped into seven different lesion classes: Melanocytic nevi (NV), Melanoma (MEL), Benign keratosis-like lesions (BKL), Basal cell carcinoma (BCC), Actinic keratoses (AKIEC), Vascular lesions (VASC), and Dermatofibroma (DF). All the images are resized to 224 × 224 pixels before input. The dataset exhibits strong class imbalance, with NV as the dominant class (6,705 images), followed by MEL (1113), BKL (1099), BCC (514), AKIEC (327), VASC (142), and DF (115). This creates an imbalance ratio of about 58:1 between the majority and minority classes. This imbalance is considered an important factor for introducing a data augmentation method and a loss function.

### 3.2 Data preprocessing and lesion-aware partitioning

The HAM10000 dataset contains multiple dermoscopic images that are captured from the same lesion under different conditions. Partitioning such data type at the image level without accounting for lesion identity introduces correlated samples across the training and validation sets. To mitigate this type of risk, a lesion-aware partitioning strategy is used throughout all experiments. This strategy ensures that no lesion identity appears simultaneously in both the training and validation sets, eliminating the possibility of identity-level data leakage and guaranteeing that all reported performance metrics reflect genuine generalization to unseen lesion identities.

#### 3.2.1 Lesion-ID deduplication

Each image in HAM10000 is associated with a unique lesion_id identifier. Lesions with exactly one associated image are first isolated to form a deduplicated subset $D_{\text{undup}}$, formally defined as shown in Equation 1:


$$\begin{array}{l} D_{\text{undup}}=\left\{l\in L||\left\{x:\text{lesion\_id}\left(x\right)=l\right\}|=1\right\} \end{array}$$ (1)

where L denotes the set of unique lesion identifiers in the dataset; this makes sure that the next train and validation split only work on lesions that do not have any duplicate views.

#### 3.2.2 Stratified train/val split

The deduplicated subset $D_{\text{undup}}$ is partitioned into training and validation sets using an 80/20 stratified split. Stratification makes sure that the proportions of all class labels are approximately maintained in both partitions as shown in Equation 2:


$$\begin{array}{l} \frac{\left|D_{\text{train}}\cap C_{k}\right|}{\left|D_{\text{train}}\right|}\approx \frac{\left|D_{\text{val}}\cap C_{k}\right|}{\left|D_{\text{val}}\right|}\approx \frac{\left|C_{k}\right|}{\left|D_{\text{undup}}\right|},\;\forall k\in \left\{1,\ldots ,7\right\} \end{array}$$ (2)

where $C_{k}$ denotes the subset of samples belonging to class *k*.

#### 3.2.3 Multi-view training set construction

To make use of the full dataset while preserving the integrity of the validation set, duplicate images of lesions already assigned to the training set are added to the final training data. Specifically for any lesion $l\in L_{\text{train}}$, all its related images across the full dataset $D_{\text{all}}$ are included as shown in Equation 3:


$$\begin{array}{l} D_{\text{train}}^{\text{final}}=D_{\text{train}}\cup \left\{x\in D_{\text{all}}|\text{lesion\_id}\left(x\right)\in L_{\text{train}}\right\} \end{array}$$ (3)

This increases training diversity by providing multiple views of the same lesion captured under different imaging conditions, while the validation set remains strictly limited to unique, deduplicated lesions. The validation set is exclusively composed of lesions whose identifiers do not appear anywhere in the training set, ensuring complete separation between training and validation partitions at the lesion identity level. This strict lesion-aware partitioning strategy eliminates any possibility of data leakage, and the consistency of results across the three-fold stratified cross-validation study presented in Section 4.7 further supports the reproducibility of the reported performance metrics.

#### 3.2.4 Dataset-specific normalization

Rather than relying on standard ImageNet statistics, per-channel mean μ_*c*_ and standard deviation σ_*c*_ are used over all the training images after resizing to 224 × 224 pixels as shown in Equation 4:


$$\begin{array}{l} \mu_{c}=\frac{1}{N}\overset{N}{\underset{i=1}{\sum }}{\bar{x}}_{i,c},\;\sigma_{c}=\sqrt{\frac{1}{N}\overset{N}{\underset{i=1}{\sum }}{\left({\bar{x}}_{i,c}-\mu_{c}\right)}^{2}} \end{array}$$ (4)

where *N* is considered as the total number of training images and ${\bar{x}}_{i,c}$ denotes the mean pixel intensity of image *i* in channel *c* ∈ {R, G, B}. This per-image approximation is computationally efficient and yields statistics that closely align with the true channel-wise mean across all pixels. Dataset-specific normalization more closely aligns the input distribution with the characteristics of dermoscopic imagery compared to natural image statistics. The complete preprocessing pipeline is summarized in Table 2.

**Table 2:** Summary of the data preprocessing pipeline applied to all images before model training and evaluation.

Step	Operation	Parameter
1	Resize	224 × 224 (bilinear interpolation)
2	Deduplication	lesion_id grouping - single-image lesions only
3	Stratified split	80/20
4	Training set construction	Multi-view enrichment via lesion_id matching
5	Normalization	Per-channel mean/std - dataset-specific

### 3.3 Data augmentation strategy

Due to the severe class imbalance in HAM10000, a carefully designed augmentation strategy is used to improve model generalization and reduce overfitting, particularly for minority classes. All augmentation techniques are applied only during the training process, and the validation set receives only resizing and normalization. A standard augmentation pipeline is applied to all seven classes during training. It includes random horizontal flipping (*p* = 0.5), random vertical flipping (*p* = 0.4), random rotation within ±20°, color jitter with brightness, contrast, and saturation perturbation factors each set to 0.25 and a hue perturbation factor of 0.12, random affine translation of ±12% with scale range [0.88, 1.12], and random erasing (*p* = 0.3). To further address class imbalance for the three most severely underrepresented classes, such as AKIEC, DF, and VASC, a strong augmentation technique is applied. During this approach, stronger horizontal flipping (*p* = 0.7), stronger vertical flipping (*p* = 0.5), increased color jitter perturbation factors of 0.3, and more aggressive affine translation of ±15% with scale range [0.85, 1.15] are applied. The full augmentation parameters for both regimes are summarized in Table 3. In addition to the geometric and color transformations, MixUp regularization is also applied stochastically during each training iteration with probability *p* = 0.4. Given two training samples (*x*_*i*_, *y*_*i*_) and (*x*_*j*_, *y*_*j*_), MixUp generates a convex combination of both samples and their label distributions, which are as shown in Equation 5:


$$\begin{array}{l} \tilde{x}=\lambda x_{i}+\left(1-\lambda \right)x_{j},\;ỹ=\lambda y_{i}+\left(1-\lambda \right)y_{j},\\ \lambda \sim \text{Beta}\left(\alpha ,\alpha \right),\alpha =0.2 \end{array}$$ (5)

**Table 3:** Comparison of standard and minority-class augmentation parameters.

Transform	Standard (All classes)	Minority classes
Horizontal flip	*p* = 0.5	*p* = 0.7
Vertical flip	*p* = 0.4	*p* = 0.5
Rotation	±20°	±20°
Color jitter	0.25 (all factors)	0.30 (all factors)
Hue jitter	0.12	0.15
Affine translation	±12%	±15%
Affine scale	[0.88, 1.12]	[0.85, 1.15]
Random erasing	*p* = 0.3	*p* = 0.3

Minority classes: AKIEC, DF, and VASC.

This interpolation promotes smoother decision boundaries and reduces overconfidence in the model's predictions. This is useful for visually similar classes such as MEL and NV.

### 3.4 Class-weighted focal loss

Standard cross-entropy loss treats all of the classes and all samples equally, so it is not suitable for heavily imbalanced datasets like HAM10000. To address this type of problem, class-weighted Focal Loss is used. In this method, class imbalance is handled using per-class weighting, and difficult classes are given high importance by considering the focal modulation factor. The standard Focal Loss introduces a modulating factor ${\left(1-p_{t}\right)}^{\gamma }$ that reduces the contribution of easy, correctly classified samples and gives more focus to hard and misclassified samples in the training part as shown in Equation 6:


$$\begin{array}{l} L_{FL}\left(p_{t}\right)=-\alpha {\left(1-p_{t}\right)}^{\gamma }\log\left(p_{t}\right) \end{array}$$ (6)

where *p*_*t*_ is considered the model's estimated probability for the ground-truth class, α represents a scalar balancing factor, and γ is the focusing parameter. In this study, we have used α = 1 and γ = 2.5. To further handle severe class imbalance in HAM10000, a per-class weight *w*_*c*_ is added to the loss formulation. For each sample *i*, the per-sample cross-entropy and the corresponding class probability are computed first, as shown in Equation 7:


$$\begin{array}{l} {\text{CE}}_{i}=\text{CrossEntropy}\left(f\left(x_{i}\right),y_{i}\right),\;p_{t,i}=e^{-{\text{CE}}_{i}} \end{array}$$ (7)

The class-weighted Focal Loss over a mini-batch of *N* samples is then defined as shown in Equation 8:


$$\begin{array}{l} L_{CFL}=\frac{1}{N}\overset{N}{\underset{i=1}{\sum }}w_{y_{i}}\cdot \alpha \cdot {\left(1-p_{t,i}\right)}^{\gamma }\cdot {\text{CE}}_{i} \end{array}$$ (8)

where *y*_*i*_ is the ground-truth class label of sample *i*, *f*(*x*_*i*_) is considered the model output logits, and *w*_*y*_*i*__ represents the class weight corresponding to the ground-truth class of sample *i*. The base class weight $w_{c}^{\text{base}}$ for each of the class *c* is computed as the inverse of its normalized frequency; the value is limited to the range [1.0, 20.0] to avoid extreme weights for very small classes:


$$\begin{array}{l} w_{c}^{\text{base}}=\text{clamp}\left(\frac{N_{\text{total}}}{K\cdot N_{c}},1.0,20.0\right) \end{array}$$ (9)

where *N*_total_ is the total number of training samples, *K* = 7 is the number of classes, and *N*_*c*_ is the number of training samples in class *c*. To further emphasize the most critically underrepresented classes, manual multipliers *m*_*c*_ are applied, and the final weight is clamped to [1.0, 18.0]:


$$\begin{array}{l} w_{c}=\text{clamp}\left(w_{c}^{\text{base}}\cdot m_{c},1.0,18.0\right) \end{array}$$ (10)

The base clamp upper bound of 20.0 in Equation 9 accommodates extreme raw-frequency imbalance ratios, whereas the tighter final clamp of 18.0 in Equation 10 serves as a safety ceiling to prevent the compounded effect of inverse-frequency weighting and manual scaling multipliers from producing excessively large weights that could destabilize gradient-based optimization. Manual scaling multipliers are applied to some of the selected classes like AKIEC (×6.5), NV (×3.8), VASC (×2.2), BKL (×1.3), and DF (×1.1). Classes BCC and MEL retain their original inverse-frequency weights; no additional scaling is applied. The multipliers are determined based on three criteria: (1) degree of underrepresentation in the training set, (2) clinical severity of misclassification, and (3) empirical observation of per-class validation performance during training. AKIEC receives the highest multiplier (×6.5) due to its premalignant clinical significance and severe underrepresentation (327 training samples). NV receives a high multiplier (×3.8) to counteract its tendency to absorb MEL predictions due to strong visual similarity between melanocytic nevi and melanoma. VASC (×2.2), BKL (×1.3), and DF (×1.1) receive progressively smaller multipliers reflecting their intermediate imbalance levels and relatively lower misclassification risk. BCC and MEL retain their base inverse-frequency weights without additional scaling, as these already provide sufficient class emphasis under the focal loss formulation. Table 4 highlights the advantages of the proposed class-weighted Focal Loss over the standard methods.

**Table 4:** Comparison of loss functions with respect to key properties for imbalanced skin lesion classification.

Property	Cross-entropy	Focal loss	Class-weighted focal loss
Easy example down-weighting	No	Yes	Yes
Class imbalance handling	No	Partial	Yes
Per-class weighting	No	No	Yes
Minority class focus	No	No	Yes

### 3.5 Heterogeneous Vision Transformer backbone selection

The proposed ensemble model is built upon three architecturally distinct Vision Transformer backbones: Swin-Tiny (22), ViT-Base (23), and DeiT-Small (24), which are selected deliberately to maximize complementary inductive bias coverage. Each backbone is initialized with ImageNet pretrained weights, its classification head is removed, and it is wrapped by the Regional Attention Wrapper described in Section 3.6. Gradient checkpointing is enabled on backbones where supported by the timm implementation to reduce GPU memory consumption during training.

#### 3.5.1 Swin-Tiny hierarchical shifted window transformer

**Figure 1:**
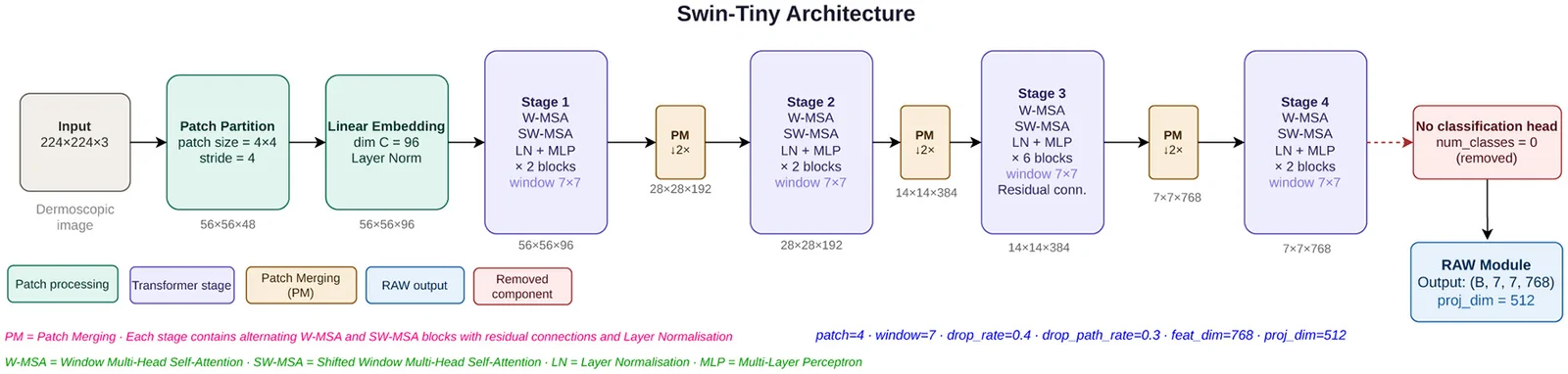
Swin-Tiny architecture with four hierarchical transformer stages. The classification head is removed and the output (*B*, 7, 7, 768) is forwarded to the RAW module.

Swin-Tiny employs a hierarchical feature extraction strategy by partitioning the input image into non-overlapping 4 × 4 patches and progressively merging them across four stages, yielding multi-scale feature representations. Unlike global self-attention, Swin-Tiny computes attention within fixed local windows of size 7 × 7, and alternates between regular window partitioning (W-MSA) and shifted window partitioning (SW-MSA) across successive blocks to enable cross-window information exchange. The shifted window self-attention is formulated as shown in Equation 11:


$$\begin{array}{l} \text{W-MSA}\left(Q,K,V\right)=\text{softmax}\left(\frac{QK^{⊤}}{\sqrt{d_{k}}}+B\right)V \end{array}$$ (11)

where *Q*, *K*, and *V* are the query, key, and value matrices, *d*_*k*_ is the key dimension, and *B* is the learnable relative position bias matrix defined within each local window. This hierarchical local attention strategy enables Swin-Tiny to efficiently capture fine-grained local texture patterns and structural features across multiple scales. These properties are particularly relevant for dermoscopic lesion characterization, where local texture irregularities are diagnostically significant. The feature dimension is *C* = 768, the projection dimension is *d*_*p*_ = 512, with a drop rate of 0.4 and a stochastic depth rate of 0.3. Figure 1 illustrates the complete Swin-Tiny architecture as employed in the proposed framework. The input dermoscopic image is divided into patches, and linear embedding is applied before passing through all four hierarchical transformer stages, each followed by patch merging to gradually reduce spatial resolution while increasing feature dimensions. The classification head is removed (num_classes=0) and the final 7 × 7 × 768 feature map is passed directly to the RAW module for spatially selective feature aggregation.

#### 3.5.2 ViT-base global self-attention transformer

**Figure 2:**
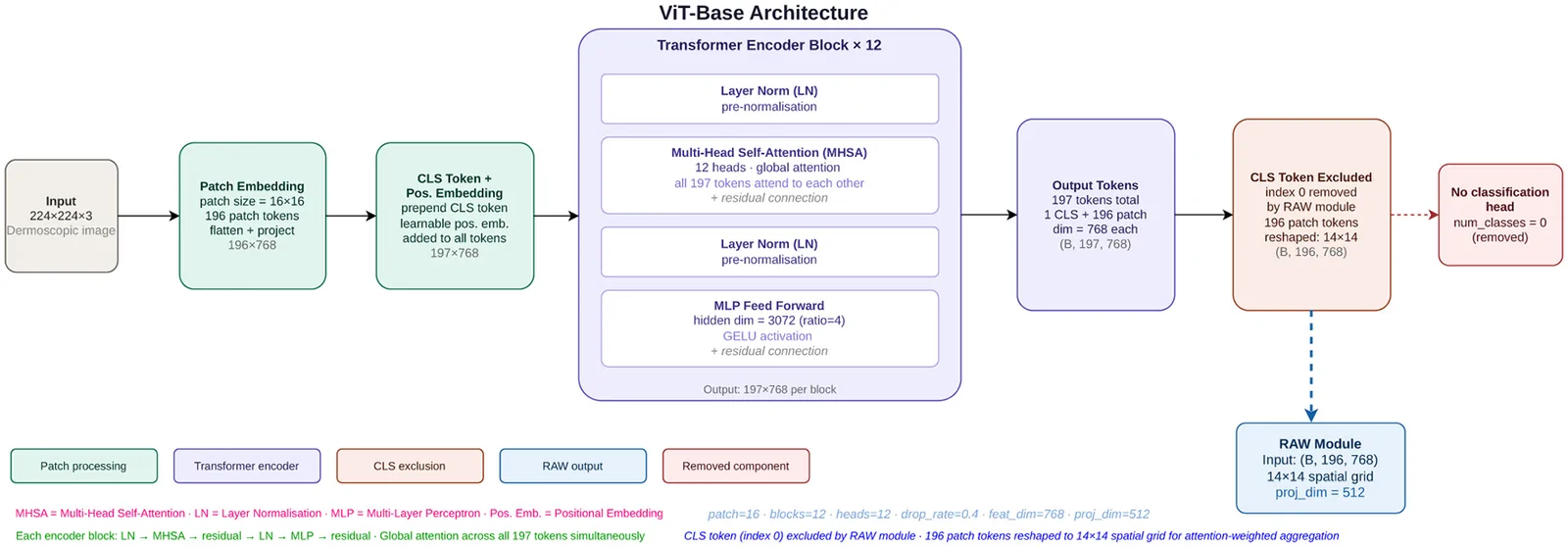
ViT-Base with 12 global self-attention encoder blocks. Output tokens (*B*, 197, 768) are reduced to (*B*, 196, 768) by removing the CLS token and passed to the RAW module (num_classes = 0).

ViT-Base treats the input image as a sequence of fixed-size non-overlapping patches of size 16 × 16, yielding 196 patch tokens for a 224 × 224 input. A learnable CLS token is prepended to the sequence, and learnable positional embeddings are added to all tokens before being passed through 12 stacked transformer encoder blocks. Unlike Swin-Tiny, ViT-Base computes self-attention globally across all tokens simultaneously, enabling the model to capture long-range spatial dependencies from the first layer. The multi-head self-attention is formulated as shown in Equation 12:


$$\begin{array}{l} \text{MHSA}\left(Q,K,V\right)=\text{softmax}\left(\frac{QK^{⊤}}{\sqrt{d_{k}}}\right)V \end{array}$$ (12)

where attention is computed across all 196 patch tokens without any local windowing constraint; this global receptive field makes ViT-Base well suited for capturing overall lesion morphology, asymmetry, and border irregularity features that span the entire dermoscopic image rather than localized regions. The RAW module excludes the CLS token, leaving 196 spatial patch tokens for attention-weighted aggregation. The feature dimension is *C* = 768, the projection dimension is *d*_*p*_ = 512 with drop rate 0.4. Figure 2 explains the complete ViT-Base architecture as employed in the proposed framework. The input image is divided into 196 patches, appended with a CLS token and learnable positional embeddings, and then processed through 12 transformer encoder blocks. The classification head is removed, and the 196 patch tokens are forwarded to the RAW module as a 14 × 14 spatial grid.

#### 3.5.3 DeiT-small data-efficient image transformer

DeiT-Small shares the same patch-based tokenization architecture as ViT, with patch size 16 × 16 yielding 196 patch tokens for a 224 × 224 input. Its key distinction lies in the training strategy: DeiT introduces a dedicated distillation token alongside the CLS token, enabling the model to learn from a CNN teacher through a knowledge distillation objective. The training loss combines standard cross-entropy with a distillation term as shown in Equation 13:


$$\begin{array}{l} L_{\text{DeiT}}=\left(1-\lambda \right)L_{\text{CE}}\left(y,ŷ\right)+\lambda \tau^{2}\text{KL}\left(\sigma \left(\frac{z_{s}}{\tau }\right)\|\sigma \left(\frac{z_{t}}{\tau }\right)\right) \end{array}$$ (13)

where *z*_*s*_ and *z*_*t*_ are the student and teacher logits respectively, τ is the distillation temperature, λ balances the two objectives, and σ(·) denotes the softmax function. It is important to note that this distillation objective governs the *pretraining* of DeiT-Small; in the present work, the pretrained DeiT-Small weights are used directly as a feature extractor without re-applying the distillation procedure. This distillation-based pretraining yields a model with stronger inductive biases inherited from the CNN teacher, producing a complementary error distribution relative to both Swin-Tiny and ViT-Base. The feature dimension is *C* = 384, the projection dimension is *d*_*p*_ = 256, with a drop rate of 0.4. DeiT-Small is used alongside Swin-Tiny and ViT-Base in the proposed ensemble model to achieve a compact and effective global attention representation. A smaller embedding dimension and fewer attention heads are being used, which yielded a distinct error distribution compared to the other two backbones. This difference is useful as the MLP meta-learner uses it during fusion. Algorithm 1 summarizes the complete forward pass of DeiT-Small as employed in the proposed framework.

Algorithm 1 DeiT-Small Forward Pass 
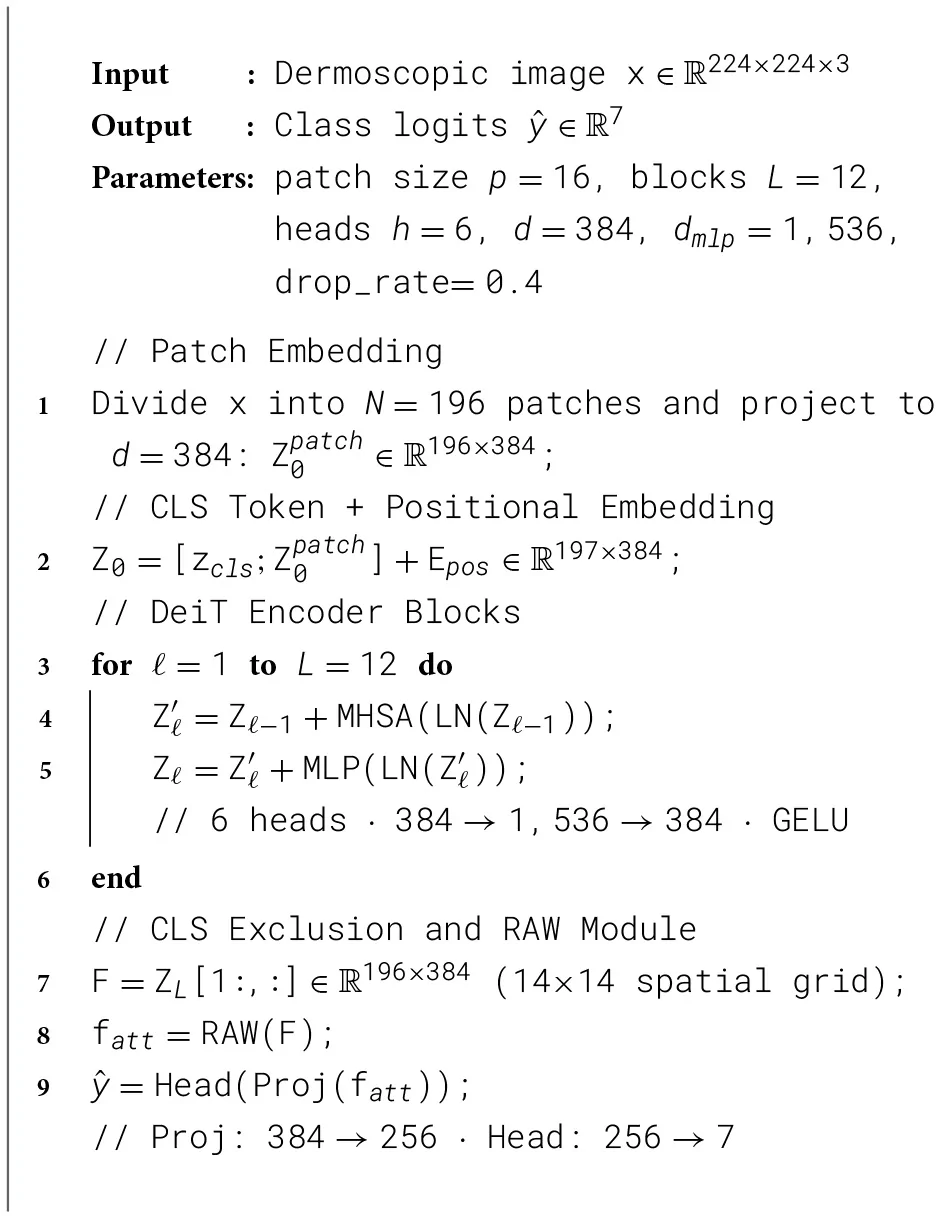

#### 3.5.4 Rationale for heterogeneous selection

The three backbones are selected to maximize architectural diversity across three key dimensions: attention locality (local window vs. global), feature hierarchy (multi-scale vs. single-scale), and training paradigm (supervised vs. distillation-based). This diversity ensures that the three models produce complementary error distributions; cases misclassified by one backbone are likely to be correctly classified by another, which is the fundamental prerequisite for an effective ensemble. A comparative summary of all three backbones is provided in Table 5.

**Table 5:** Comparison of the three Vision Transformer backbones used in the proposed heterogeneous ensemble.

Backbone	Pretrain	Attention	*C*	*d*_*p*_	Drop rate	Drop path	Output shape
Swin-Tiny	IN-1K	Local window	768	512	0.4	0.3	(*B*, 7, 7, 768)
ViT-Base	IN-1K	Global	768	512	0.4	^*^	(*B*, 197, 768)
DeiT-Small	IN-1K	Global	384	256	0.4	^*^	(*B*, 197, 384)

^*^Drop-path regularization is not applied in this configuration.

### 3.6 Regional Attention Wrapper (RAW)

Standard feature aggregation strategies such as CLS token extraction and global mean pooling treat all spatial regions of the feature map equally, without accounting for the spatial non-uniformity of dermoscopic lesions. In dermoscopy, diagnostically relevant features such as irregular borders, atypical pigmentation networks, and vascular structures are spatially localized and occupy only a fraction of the image. A pooling strategy that weights all regions equally risks diluting these discriminative features with uninformative background skin regions. To address this, the Regional Attention Wrapper is introduced as a unified, learnable spatial attention module applied consistently across all three backbones.

#### 3.6.1 Feature tensor unification

The three backbone architectures produce feature tensors of different shapes. To apply a uniform attention mechanism across all three, the RAW module first unifies these tensors into a common format F ∈ ℝ^*B*×*M*×*C*^, where *B* is the batch size, *M* is the number of spatial tokens, and *C* is the feature dimension. The unification rules are as follows. For ViT-Base and DeiT-Small, the backbone returns a 3D tensor of shape (*B, N, C*) where the first token is the CLS token. The CLS token is excluded, and the remaining *N* − 1 patch tokens are retained as shown in Equation 14:


$$\begin{array}{l} \text{F}=\text{feats}\left[:,1:,:\right]\in {\mathbb{R}}^{B\times 196\times C} \end{array}$$ (14)

For Swin-Tiny, the backbone returns a 4D tensor of shape (*B, H, W, C*), which is reshaped along the spatial dimensions as shown in Equation 15:


$$\begin{array}{l} \text{F}=\text{reshape}\left(\text{feats},B,H\times W,C\right)\in {\mathbb{R}}^{B\times 49\times 768} \end{array}$$ (15)

In the edge case where the backbone returns a 2D global feature vector of shape (*B, C*), it is unsqueezed to (*B*, 1, *C*) to maintain interface consistency.

#### 3.6.2 Attention scoring and weighted aggregation

Given the unified spatial token tensor F ∈ ℝ^*B*×*M*×*C*^, a learnable linear layer ${\text{W}}_{a}\in {\mathbb{R}}^{C\times 1}$ computes a scalar importance score for each spatial token as shown in Equation 16:


$$\begin{array}{l} s_{i}={\text{W}}_{a}{\text{f}}_{i}+b_{a},\;i=1,\ldots ,M \end{array}$$ (16)

where ${\text{f}}_{i}\in {\mathbb{R}}^{C}$ is the feature vector of the *i*-th spatial token. The scores are normalized across all *M* tokens via softmax to produce attention weights as shown in Equation 17:


$$\begin{array}{l} \alpha_{i}=\frac{\exp\left(s_{i}\right)}{\sum_{j=1}^{M}\exp\left(s_{j}\right)},\;\overset{M}{\underset{i=1}{\sum }}\alpha_{i}=1 \end{array}$$ (17)

The attended feature vector is then computed as a weighted sum over all spatial tokens as shown in Equation 18:


$$\begin{array}{l} {\text{f}}_{\text{att}}=\overset{M}{\underset{i=1}{\sum }}\alpha_{i}{\text{f}}_{i}\in {\mathbb{R}}^{C} \end{array}$$ (18)

This formulation allows the model to focus selectively on the most diagnostically relevant spatial regions while suppressing uninformative background tokens.

#### 3.6.3 Projection and classification head

The attended feature vector f_att_ is first projected to a lower-dimensional space via a linear projection layer ${\text{W}}_{p}\in {\mathbb{R}}^{C\times d_{p}}$ as shown in Equation 19:


$$\begin{array}{l} {\text{f}}_{\text{proj}}={\text{W}}_{p}{\text{f}}_{\text{att}}\in {\mathbb{R}}^{d_{p}} \end{array}$$ (19)

The projected feature is then passed through a two-stage classification head as shown in Equation 20:


$$\begin{array}{l} ŷ={\text{W}}_{3}\text{ReLU}\left({\text{W}}_{2}\text{BN}\left(\text{ReLU}\left({\text{W}}_{1}\text{BN}\left({\text{f}}_{\text{proj}}\right)\right)\right)\right) \end{array}$$ (20)

where the head comprises: Linear(*d*_*p*_ → 512) → BN → ReLU → Dropout(0.4) → Linear(512 → 256) → BN → ReLU → Dropout(0.3) → Linear(256 → 7), where *d*_*p*_ takes the value 512 for Swin-Tiny and ViT-Base, and 256 for DeiT-Small. The RAW configuration for each backbone is summarized in Table 6.

**Table 6:** Regional Attention Wrapper configuration for each backbone.

Backbone	*C*	*d*_*p*_	*M*	Dropout 1	Dropout 2
Swin-Tiny	768	512	49	0.4	0.3
ViT-Base	768	512	196	0.4	0.3
DeiT-Small	384	256	196	0.4	0.3

#### 3.6.4 Dual role of attention weights

The attention weights **α** ∈ ℝ^*M*^ serve a dual purpose in the proposed framework. During forward inference, they drive the spatially selective feature aggregation described above. For explainability analysis, the weights are reshaped into a 2D spatial map of size 7 × 7 for Swin-Tiny and 14 × 14 for ViT-Base and DeiT-Small, corresponding to the spatial token grid of each backbone. These maps directly reflect which spatial regions of the input image the model weighted most heavily during classification, requiring no *post-hoc* gradient computation. The ensemble attention map is obtained by averaging the attention maps across all three backbones, as described in Section 3.9. The dual role of the RAW attention weights α for each backbone is summarized in Table 7.

**Table 7:** Dual role of RAW attention weights **α** for each backbone.

Backbone	M	C	Inference role	Spatial map	Upsampled to
Swin-Tiny	49	768	$f_{\text{att}}=\overset{M}{\underset{i=1}{\sum }}\alpha_{i}f_{i}$	7 × 7	224 × 224
ViT-Base	196	768	$f_{\text{att}}=\overset{M}{\underset{i=1}{\sum }}\alpha_{i}f_{i}$	14 × 14	224 × 224
DeiT-Small	196	384	$f_{\text{att}}=\overset{M}{\underset{i=1}{\sum }}\alpha_{i}f_{i}$	14 × 14	224 × 224

Ensemble: $\bar{\alpha }=\frac{1}{3}\overset{3}{\underset{k=1}{\sum }}\alpha^{\left(k\right)}$, averaged across all three backbones.

### 3.7 MLP meta-learner for ensemble fusion

#### 3.7.1 Motivation and stacking protocol

Simple ensemble fusion strategies such as soft voting and naive averaging treat all backbone outputs uniformly, assigning equal importance to each model regardless of its confidence or class-specific reliability. Such strategies fail to exploit the learned inter-model complementarity that arises from the heterogeneous backbone selection. To address this, a three-layer MLP is employed as a meta-learner through a stacking protocol, a second-level learning strategy in which a separate model is trained to fuse the outputs of the base learners. The meta-learner learns to weight and combine backbone predictions in a non-linear, class-aware manner.

#### 3.7.2 Stacking input construction

After backbone training converges, each backbone generates a 7-dimensional softmax probability vector for every input image as shown in Equation 21:


$$\begin{array}{r} {\text{p}}^{\left(k\right)}=\text{softmax}\left(f_{k}\left(x\right)\right)\in {\mathbb{R}}^{7},\\ k\in \left\{\text{Swin-Tiny, ViT-Base, DeiT-Small}\right\} \end{array}$$ (21)

where *f*_*k*_(*x*) denotes the logit output of the *k*-th backbone for input *x*. The three probability vectors are concatenated in fixed order to form the 21-dimensional meta-input vector:


$$\begin{array}{l} \text{z}=\left[{\text{p}}^{\left(\text{Swin}\right)}\|{\text{p}}^{\left(\text{ViT}\right)}\|{\text{p}}^{\left(\text{DeiT}\right)}\right]\in {\mathbb{R}}^{21} \end{array}$$ (22)

This concatenated representation encodes not only the individual class confidence of each backbone but also their relative agreement and disagreement patterns across all seven classes, information that naive averaging discards.

#### 3.7.3 Meta-learner architecture

The meta-learner processes the 21-dimensional input through three fully connected layers with batch normalization, non-linear activation, and dropout regularization as shown in Equation 23:


$$\begin{array}{l} {ŷ}_{\text{ens}}={\text{W}}_{3}\text{ReLU}\left({\text{W}}_{2}\text{ReLU}\left(\text{BN}\left({\text{W}}_{1}\text{z}+{\text{b}}_{1}\right)\right)\right) \end{array}$$ (23)

where the full layer sequence is: Linear(21 → 64) → BN → ReLU → Dropout(0.3) → Linear(64 → 32) → ReLU → Linear(32 → 7). Dropout with rate 0.3 is applied after the first ReLU activation, as indicated in Table 8. The final output ${ŷ}_{\text{ens}}\in {\mathbb{R}}^{7}$ contains the ensemble logits, which are passed through softmax to obtain the final class probabilities. The complete layer configuration is summarized in Table 8.

**Table 8:** Meta-learner layer configuration.

Layer	Type	Input dim	Output dim	BN	Activation	Dropout
1	Linear	21	64	Yes	ReLU	0.3
2	Linear	64	32	No	ReLU	–
3	Linear	32	7	No	–	–

#### 3.7.4 Meta-learner training

The meta-learner is trained using the Adam optimizer with a learning rate of 1 × 10^−3^ and weight decay of 1 × 10^−4^. The training objective is the standard cross-entropy loss over the stacking dataset as shown in Equation 24:


$$\begin{array}{l} L_{\text{meta}}=-\frac{1}{N}\overset{N}{\underset{i=1}{\sum }}\overset{7}{\underset{c=1}{\sum }}y_{i,c}\log{\hat{p}}_{i,c} \end{array}$$ (24)

where *y*_*i,c*_ is the one-hot ground-truth label and ${\hat{p}}_{i,c}$ is the predicted probability for class *c* of sample *i*. Training proceeds for 50 epochs with batch size 32. All backbone weights are frozen during meta-learner training; only the meta-learner parameters are updated. Following training, the meta-learner weights are fixed, and the model is used exclusively in inference mode for all subsequent evaluation and explainability analyses.

### 3.8 Training protocol

#### 3.8.1 Phase 1 - joint backbone training

All three RAW-wrapped backbones are trained jointly in an end-to-end manner for up to 50 epochs. Each backbone is assigned a dedicated AdamW optimizer with individualized learning rates reflecting their architectural complexity and pretrained weight sensitivity: Swin-Tiny at 5 × 10^−5^, ViT-Base at 3 × 10^−5^, and DeiT-Small at 4 × 10^−5^. A weight decay of 5 × 10^−4^ is applied uniformly across all three optimizers to regularize the learned parameters. The learning rate for each backbone is annealed using a cosine schedule over the full training horizon as shown in Equation 25:


$$\begin{array}{l} \eta_{t}=\eta_{\min}+\frac{1}{2}\left(\eta_{\max}-\eta_{\min}\right)\left(1+\cos\frac{t\pi }{T_{\max}}\right) \end{array}$$ (25)

where η_max_ is the initial learning rate, $\eta_{\min}=1\times 10^{-6}$ is the minimum learning rate, *t* is the current epoch, and *T*_max_ = 50 is the total number of epochs. To stabilize training under the memory constraints of the NVIDIA Tesla T4 GPU, gradient accumulation is employed over six consecutive steps before a parameter update is performed. With a per-step batch size of 6, the effective batch size is as shown in Equation 26:


$$\begin{array}{l} B_{\text{eff}}=B_{\text{step}}\times N_{\text{accum}}=6\times 6=36 \end{array}$$ (26)

Gradient norms are clipped to a maximum of 0.5 at each update step to prevent gradient explosion. Automatic Mixed Precision training is used throughout to reduce memory consumption and accelerate computation. To prevent overfitting, early stopping is applied based on validation accuracy. Training is terminated if no improvement greater than δ = 3 × 10^−4^ is observed over *p* = 12 consecutive epochs, subject to a minimum training duration of 25 epochs as shown in Equation 27:


$$\begin{array}{l} \text{stop if}\Delta {\text{Acc}}_{\text{val}}<\delta \text{ for }p\text{ consecutive epochs},\\ \delta =3\times 10^{-4},p=12,t\geq 25 \end{array}$$ (27)

The model checkpoint achieving the highest validation accuracy is saved and restored at the end of training.

The hyperparameter configuration was determined through a combination of established best practices from the Vision Transformer literature and systematic empirical validation on the HAM10000 validation set. The rationale for each key hyperparameter is described as follows.

Learning rates: individualized learning rates were assigned to each backbone reflecting their architectural complexity and pretrained weight sensitivity. ViT-Base, as the largest model with 86M parameters, was assigned the lowest learning rate of 3 × 10^−5^ to prevent disruption of its pretrained weight distribution. DeiT-Small was assigned 4 × 10^−5^, reflecting its intermediate sensitivity due to distillation-based pretraining. Swin-Tiny was assigned 5 × 10^−5^, consistent with its hierarchical local attention architecture, which adapts more readily to domain-specific features.

Weight decay: a uniform weight decay of 5 × 10^−4^ was applied across all three backbone optimizers, providing sufficient ℓ_2_ regularization to prevent overfitting while avoiding excessive parameter shrinkage that could impair the pretrained feature representations.

Cosine annealing schedule: the cosine annealing learning rate schedule was selected due to its smooth and gradual learning rate reduction, which improves final model convergence in transformer-based architectures. The minimum learning rate was set to $\eta_{\min}=1\times 10^{-6}$ to prevent complete learning rate collapse in the final training epochs.

Gradient accumulation and batch size: a per-step batch size of 6 with gradient accumulation over six steps, yielding an effective batch size of 36, was adopted due to the GPU memory constraints of the NVIDIA Tesla T4, ensuring stable gradient estimates within the available memory budget.

Gradient clipping: gradient norms were clipped to a maximum of 0.5 to prevent gradient explosion when jointly training three large transformer backbones with a shared loss function.

Early stopping: a patience of 12 epochs with a minimum improvement threshold of δ = 3 × 10^−4^ and a minimum training duration of 25 epochs was adopted to prevent premature termination while avoiding overfitting.

Meta-learner hyperparameters: the meta-learner was trained using the Adam optimizer with learning rate 1 × 10^−3^ and weight decay 1 × 10^−4^, following established practice for shallow MLP classifiers.

#### 3.8.2 Phase 2 - meta-learner training

Following backbone training, the meta-learner is trained as described in Section 3.7 using the Adam optimizer with learning rate 1 × 10^−3^, weight decay 1 × 10^−4^, cross-entropy loss, batch size 32, and 50 epochs. The complete hyperparameter configuration for both training phases is summarized in Table 9.

**Table 9:** Complete training hyperparameter summary for both phases.

Hyperparameter	Phase 1 (Backbones)	Phase 2 (Meta-learner)
Optimiser	AdamW	Adam
LR - Swin-Tiny	5 × 10^−5^	–
LR - ViT-Base	3 × 10^−5^	–
LR - DeiT-Small	4 × 10^−5^	–
LR - Meta-Learner	-	1 × 10^−3^
Weight decay	5 × 10^−4^	1 × 10^−4^
LR schedule	Cosine annealing	–
η_min_	1 × 10^−6^	–
Batch size	6 (effective 36)	32
Gradient accumulation	6 steps	–
Gradient clipping	0.5	–
AMP	Yes	No
Max epochs	50	50
Early stopping patience	12	–
Early stopping δ	3 × 10^−4^	–
Min epochs before stop	25	–
Hardware	NVIDIA Tesla T4	

### 3.9 Explainability and statistical validation framework

All explainability analyses are performed on a held-out clean validation subset re-split from deduplicated lesions. Three visualization outputs are produced per sample per method: the original image, a standalone heatmap, and an image overlay.

#### 3.9.1 Regional attention map visualization

The spatial attention weights **α**^(*k*)^ ∈ ℝ^*H*×*W*^ from each backbone's RAW module are extracted directly during inference without any additional gradient computation. The three per-backbone attention maps are averaged to produce an ensemble-level attention map as shown in Equation 28:


$$\begin{array}{l} \bar{\alpha }=\frac{1}{3}\overset{3}{\underset{k=1}{\sum }}\alpha^{\left(k\right)},\;\bar{\alpha }\in {\mathbb{R}}^{H\times W} \end{array}$$ (28)

The resulting map is bilinearly upsampled to 224 × 224 pixels and rendered using the JET colormap, where warmer colors indicate regions of higher attention weight. Since the attention weights are a direct byproduct of the RAW module's forward pass, this visualization method requires no *post-hoc* gradient computation and introduces no additional computational overhead. The integration of hybrid attention mechanisms combining local and global feature extraction has been demonstrated to enhance classification performance in medical imaging tasks, as evidenced by recent work in colon cancer detection.

#### 3.9.2 Ensemble GradCAM++

GradCAM++ is applied independently to each backbone at its final normalization layer. For ViT-Base and DeiT-Small, the patch token sequence is reshaped into a 14 × 14 spatial grid prior to gradient accumulation. The GradCAM++ importance weight for the *k*-th feature map channel is computed as shown in Equation 29:


$$\begin{array}{l} \alpha_{k}^{c}=\frac{\frac{\partial^{2}S^{c}}{\partial {\left(A_{k}^{c}\right)}^{2}}}{2\frac{\partial^{2}S^{c}}{\partial {\left(A_{k}^{c}\right)}^{2}}+\underset{i,j}{\sum }A_{k}^{c}\cdot \frac{\partial^{3}S^{c}}{\partial {\left(A_{k}^{c}\right)}^{3}}} \end{array}$$ (29)

where *S*^*c*^ is the class score for target class *c* and $A_{k}^{c}$ is the *k*-th activation map. The ensemble GradCAM++ map is obtained by averaging the per-backbone saliency maps as shown in Equation 30:


$$\begin{array}{l} G_{\text{ens}}=\frac{1}{3}\overset{3}{\underset{k=1}{\sum }}\text{ReLU}\left(\underset{c}{\sum }\alpha_{k}^{c}\cdot A_{k}^{c}\right) \end{array}$$ (30)

The resulting map is normalized to [0, 1], rendered using the JET colormap, and superimposed on the original image.

#### 3.9.3 SHAP GradientExplainer

SHAP attribution is computed for the complete ensemble pipeline through a unified end-to-end differentiable ensemble module that encapsulates all three RAW-wrapped backbones and the meta-learner. The GradientExplainer is initialized with a background reference set of 50 randomly sampled validation images. The background reference set of 50 samples is constructed through stratified sampling across all seven lesion classes, ensuring that the marginal reference distribution is representative of the full validation set rather than being dominated by the majority NV class. This size is consistent with established SHAP GradientExplainer practice for image classification tasks, where background sets in the range of 50–100 samples produce stable expected gradient estimates. This choice additionally balances attribution reliability against the memory and computational constraints of the NVIDIA Tesla T4 GPU, where larger background sets significantly increase per-image attribution computation time. For each test image, SHAP values are computed with respect to the predicted class using the expected gradient formulation as shown in Equation 31:


$$\begin{array}{l} \phi_{j}={𝔼}_{x^{\prime }\sim D_{\text{bg}}}\left[\nabla_{x_{j}}f\left(x\right)\cdot \left(x_{j}-x_{j}^{\prime }\right)\right] \end{array}$$ (31)

where *x*′ is a background reference sample drawn from $D_{\text{bg}}$, *f*(*x*) is the ensemble model output, and ϕ_*j*_ is the attribution for input pixel *j*. The three-channel attribution tensor is averaged over color channels to produce a 2D attribution map as shown in Equation 32:


$$\begin{array}{l} \Phi \left(i,j\right)=\frac{1}{3}\overset{3}{\underset{c=1}{\sum }}|\phi_{c,i,j}| \end{array}$$ (32)

The attribution map is smoothed using a Gaussian filter with kernel size 11 × 11 and normalized symmetrically using the 99th percentile of absolute attribution magnitude as shown in Equation 33:


$$\begin{array}{l} \hat{\Phi }=\text{clip}\left(\frac{\Phi }{P_{99}\left(\left|\Phi \right|\right)},-1,1\right) \end{array}$$ (33)

The normalized map is rendered using a diverging RdBu_r colormap, where red regions indicate positive class-confirming attributions and blue regions indicate suppressive attributions.

#### 3.9.4 t-SNE feature space analysis

To visualize the class separability achieved by each model, t-SNE projections are computed for all four models. For individual backbones, feature vectors are extracted from each backbone and spatially mean-pooled to obtain a fixed-dimensional feature vector. For the ensemble, the 21-dimensional concatenated softmax probability vector z defined in Equation 22 serves directly as the feature representation, reflecting the meta-learner's decision space. The t-SNE objective minimizes the Kullback–Leibler divergence between the high-dimensional and low-dimensional pairwise similarity distributions as shown in Equation 34:


$$\begin{array}{l} L_{\text{t-SNE}}=\underset{i}{\sum }\text{KL}\left(P_{i}\|Q_{i}\right)=\underset{i}{\sum }\underset{j}{\sum }p_{j|i}\log\frac{p_{j|i}}{q_{j|i}} \end{array}$$ (34)

where *p*_*j*|*i*_ and *q*_*j*|*i*_ are the conditional similarities in the high-dimensional and low-dimensional spaces, respectively. Projections are computed with perplexity = 30, 1,000 iterations, and a fixed random seed of 42 to ensure full reproducibility of the 2D visualizations across runs. The projections are color-coded by class to assess intra-class compactness and inter-class separability.

#### 3.9.5 McNemar's statistical significance test

To validate that observed performance differences between the proposed model and each individual backbone are statistically significant rather than attributable to sampling variability, McNemar's test is applied to all six pairwise model comparisons on the shared test set. For each pair of models (*A, B*), a 2 × 2 contingency table is constructed from their binary correctness vectors as shown in Equation 35:


$$\begin{array}{l} \left(\begin{array}{cc} a & b\\ c & d \end{array}\right) \end{array}$$ (35)

where *a* = both correct; *b* = *A* correct, *B* wrong; *c* = *A* wrong, *B* correct; *d* = both wrong. The test statistic with Yates' continuity correction is as shown in Equation 36:


$$\begin{array}{l} \chi^{2}=\frac{{\left(|b-c|-1\right)}^{2}}{b+c} \end{array}$$ (36)

A *p*-value below α = 0.05 (corresponding to $\chi_{\text{critical}}^{2}=3.841$) is considered statistically significant. The six pairwise comparisons evaluated are summarized in Table 10.

**Table 10:** Pairwise model comparisons evaluated using McNemar's statistical significance test.

Comparison	Model A	Model B
1	Proposed model	Swin-Tiny
2	Proposed model	ViT-Base
3	Proposed model	DeiT-Small
4	Swin-Tiny	ViT-Base
5	Swin-Tiny	DeiT-Small
6	ViT-Base	DeiT-Small

### 3.10 Integrated system architecture and inference pipeline

#### 3.10.1 End-to-end architecture overview

**Figure 3:**
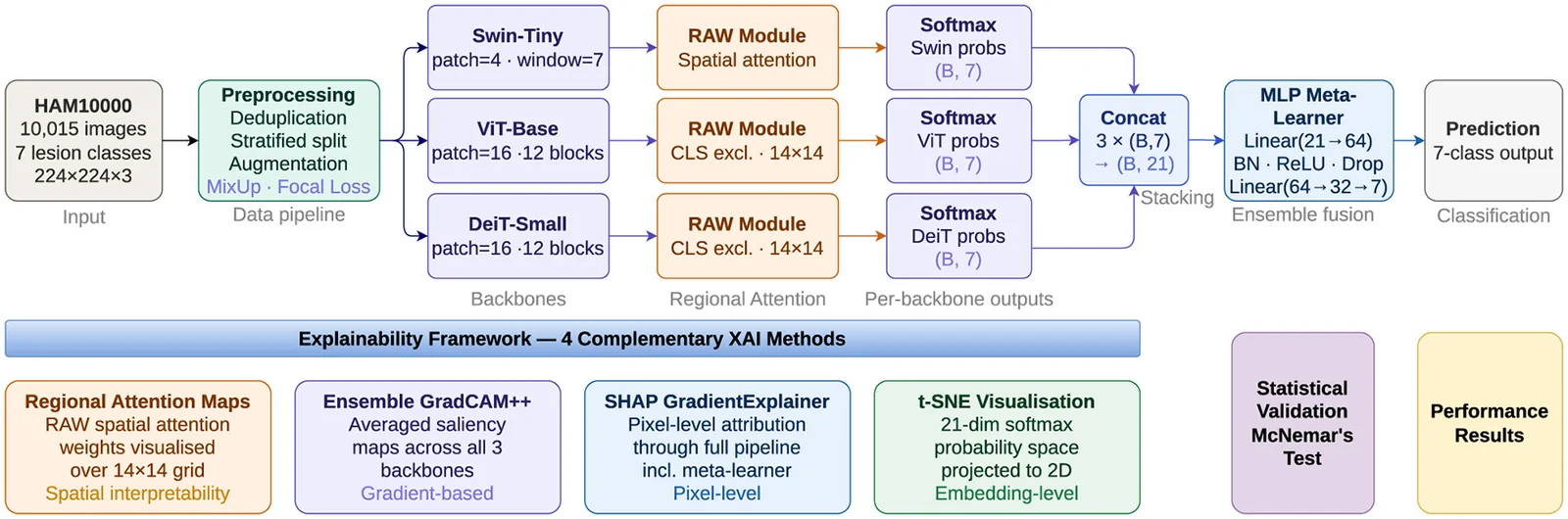
Proposed heterogeneous Vision Transformer ensemble framework for dermoscopic skin lesion classification with multi-method explainability.

The proposed framework integrates all previously described components into a unified two-phase system for dermoscopic skin lesion classification. As illustrated in Figure 3, the architecture consists of three parallel processing streams, each comprising a pretrained Vision Transformer backbone wrapped by the RAW module. Each stream independently processes the input dermoscopic image and produces a 7-dimensional softmax probability vector. These three probability vectors are concatenated to form the 21-dimensional meta-input vector, which is subsequently processed by the MLP meta-learner to produce the final class prediction. The XAI framework operates in parallel with the inference pipeline, extracting attention maps, GradCAM++ saliency maps, and SHAP attributions from the same forward and backward passes used for classification.

The design follows a two-phase training paradigm: in Phase 1, the three RAW-wrapped backbones are trained jointly using the class-weighted Focal Loss with class-adaptive augmentation and MixUp regularization. This decoupled training strategy ensures that each component is optimized for its specific role without interference.

#### 3.10.2 Inference pipeline

At inference time, a single dermoscopic image is processed through the sequence of operations described in Algorithm 2.

Algorithm 2 Inference pipeline of the proposed ensemble 
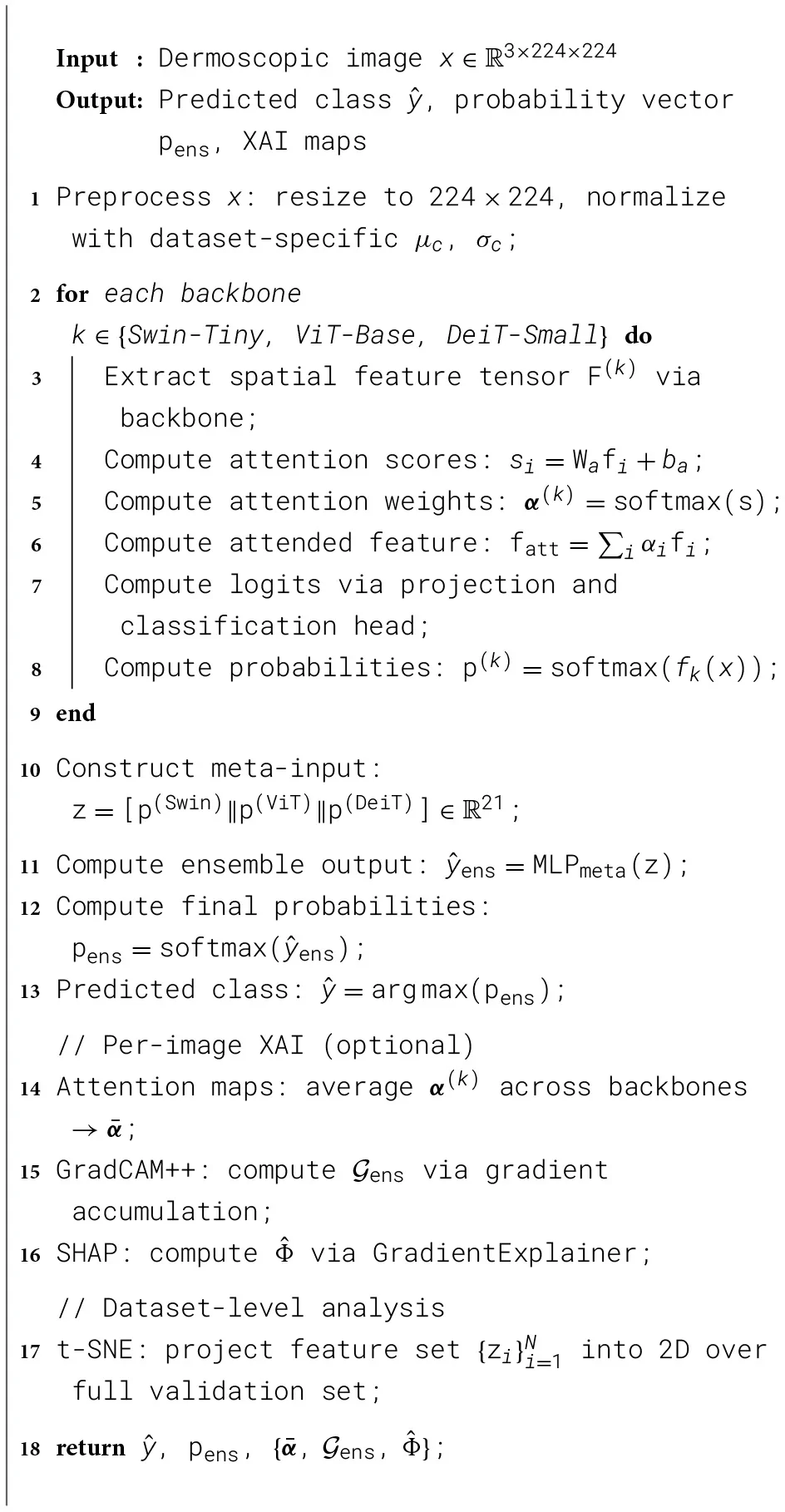

#### 3.10.3 Complementary feature representation

The three backbones contribute complementary feature representations that collectively span a broader diagnostic feature space than any single model. Swin-Tiny, through its hierarchical shifted window attention, captures fine-grained local texture patterns such as pigmentation network structure, follicular openings, and surface irregularities at multiple scales. ViT-Base, operating with global self-attention from the first layer, captures long-range spatial relationships including overall lesion shape, border asymmetry, and global color distribution. DeiT-Small, trained with CNN distillation, inherits inductive biases from convolutional networks while retaining transformer-level feature abstraction, providing a representation that bridges local and global perspectives. The meta-learner exploits these complementary representations by learning to resolve inter-model disagreements in a class-aware manner. When all three backbones agree on a prediction, the meta-learner reinforces the consensus. When disagreement occurs, as is common for visually ambiguous classes such as MEL vs. NV, the meta-learner has learned which backbone's confidence is most reliable for that specific class combination, effectively acting as a learned arbitrator.

#### 3.10.4 Computational complexity and parameter summary

The total parameter count of the proposed framework is the sum of the three RAW-wrapped backbone parameter counts and the meta-learner as shown in Equation 37:


$$\begin{array}{l} N_{\text{total}}=\overset{3}{\underset{k=1}{\sum }}N_{k}^{\text{backbone+RAW}}+N_{\text{meta}} \end{array}$$ (37)

where $N_{k}^{\text{backbone+RAW}}$ includes all parameters of the *k*-th backbone and its associated RAW projection and classification head. The parameter breakdown is summarized in Table 11.

**Table 11:** Parameter count and input/output shape summary for each component of the proposed framework.

Component	Parameters	Input shape	Output shape
Swin-Tiny + RAW	~28M	(*B*, 3, 224, 224)	(*B*, 7)
ViT-Base + RAW	~86M	(*B*, 3, 224, 224)	(*B*, 7)
DeiT-Small + RAW	~22M	(*B*, 3, 224, 224)	(*B*, 7)
MLP Meta-Learner	~4K	(*B*, 21)	(*B*, 7)
Total	~136M	–	–

The complete framework requires approximately 7–8 h of training time on an NVIDIA Tesla T4 GPU, reflecting the computational cost of jointly training three large-scale Vision Transformer backbones with the RAW module across 50 epochs using gradient accumulation.

#### 3.10.5 Multi-method explainability integration

The four XAI methods employed in this work are designed to be complementary rather than redundant, each operating at a different level of abstraction and providing a distinct perspective on the model's decision-making process. The RAW attention maps provide spatial focus information directly from the model's internal attention mechanism, requiring no gradient computation. GradCAM++ provides gradient-weighted spatial localization at the final feature layer of each backbone, highlighting the most discriminative image regions for the predicted class. SHAP GradientExplainer operates at the input pixel level, providing signed attributions that distinguish between class-confirming and class-suppressing regions. t-SNE operates at the embedding level, providing a global view of class separability and cluster structure across the validation set. Together, these four methods provide a multi-level, multi-perspective explainability framework that addresses both spatial localization and feature-space interpretability requirements. A comparative summary of all four methods is provided in Table 12.

**Table 12:** Comparison of XAI methods employed in the proposed framework.

Method	Level	Output	Gradient	Colormap
Attention Maps	Token/spatial	*H* × *W* heatmap	No	JET
GradCAM++	Activation map	*H* × *W* saliency	Yes	JET
SHAP	Input pixel	*H* × *W* attribution	Yes	RdBu_r
t-SNE	Embedding	2D scatter plot	No	Class-coded

## 4 Results

This section presents the experimental evaluation of the proposed heterogeneous Vision Transformer ensemble framework on the HAM10000 dataset, covering experimental setup, training dynamics, overall and per-class classification performance, confusion matrix analysis, ROC curve and AUC analysis, statistical significance validation, and explainability analysis.

### 4.1 Experimental setup

**Figure 4:**
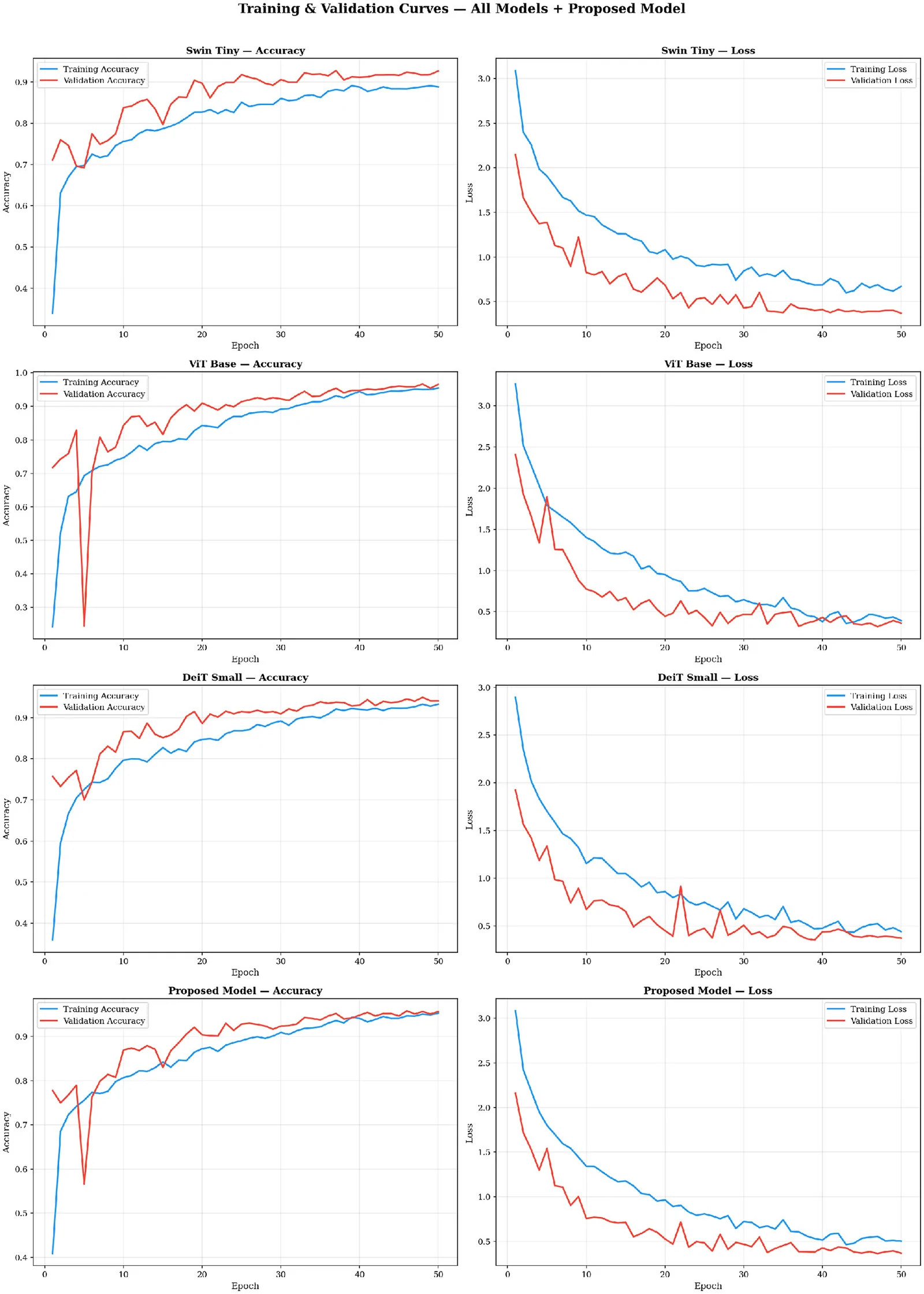
Training and validation accuracy and loss curves for all four models.

All experiments are conducted on the HAM10000 dataset comprising 10,015 dermoscopic images across seven lesion classes. Following the lesion-aware partitioning strategy, the dataset is divided into a training set of 5,070 images and a validation set of 1,103 images using stratified sampling to preserve class proportions. All images are resized to 224 × 224 pixels and normalized using dataset-specific per-channel statistics. All models are implemented in PyTorch and trained on an NVIDIA Tesla T4 GPU using Automatic Mixed Precision. The proposed framework is compared against its three individual backbone components, Swin-Tiny, ViT-Base, and DeiT-Small, each trained under identical conditions to ensure a fair comparison. Performance is evaluated using overall accuracy, weighted precision, weighted recall, weighted F1-score, per-class precision, recall, and F1-score, macro-averaged AUC, and McNemar's statistical significance test. The minority classes such as DF, VASC, and AKIEC have limited validation set sizes. However, the lesion-aware partitioning strategy, combined with class-adaptive augmentation, MixUp regularization, dropout, and early stopping, collectively mitigates the risk of overfitting. The consistent pattern of validation accuracy exceeding training accuracy throughout training in Figure 4 further confirms the absence of overfitting in the proposed framework.

### 4.2 Training dynamics

The proposed framework is trained for the full 50 epochs without early stopping triggering, indicating stable and continued improvement throughout training. The best backbone checkpoint is saved at epoch 46 with a validation accuracy of 0.9583. The MLP meta-learner converges rapidly, reaching its lowest training loss of 0.0851 at epoch 30, with the loss stabilizing between 0.0851 and 0.0884 over the remaining epochs, indicating robust convergence without overfitting.

Figure 4 presents the training and validation accuracy and loss curves for all four models. All three backbones exhibit a characteristic pattern in which validation accuracy consistently exceeds training accuracy throughout training, attributable to the class-adaptive augmentation and MixUp regularization applied exclusively during training. Swin-Tiny converges smoothly and steadily across all 50 epochs with no significant instability. DeiT-Small demonstrates the fastest initial convergence among the three backbones, reaching approximately 0.85 validation accuracy by epoch 10. ViT-Base exhibits a notable transient dip at epoch 5 where validation accuracy drops to 0.2439, attributable to the interaction between the pretrained ViT-Base weights and the cosine annealing learning rate schedule in the absence of a dedicated warmup phase. As a large global self-attention model with 86M parameters initialized from ImageNet pretrained weights, ViT-Base is particularly sensitive to learning rate perturbations in the early epochs. The cosine annealing schedule begins from the initial learning rate of 3 × 10^−5^ without a warmup phase, causing a transient disruption to the pretrained weight distribution before the model successfully adapts to the dermoscopic feature space, followed by rapid recovery and strong convergence thereafter. The proposed model inherits this transient behavior at epoch 5 but recovers fully by epoch 8 and achieves consistently superior validation accuracy from epoch 18 onwards.

### 4.3 Overall classification performance

The proposed heterogeneous ensemble achieves an overall accuracy of 98.37%, a weighted precision of 98.44%, a weighted recall of 98.37%, and a weighted F1-score of 98.39% on the validation set, outperforming all three individual backbones across all metrics. Among the individual backbones, ViT-Base achieves the highest accuracy at 95.83%, followed by DeiT-Small at 94.56% and Swin-Tiny at 92.38%. The ensemble improves upon the best individual backbone (ViT-Base) by 2.54 percentage points in accuracy, demonstrating that the heterogeneous diversity of the three backbones and the learned MLP meta-learner fusion contribute measurable gains beyond any single model. The complete performance summary is presented in Table 13.

**Table 13:** Overall classification performance of all four models.

Model	Accuracy	Precision	Recall	F1-score
Swin-Tiny	0.9238	0.9449	0.9238	0.9289
ViT-Base	0.9583	0.9611	0.9583	0.9589
DeiT-Small	0.9456	0.9553	0.9456	0.9477
**Proposed model**	**0.9837**	**0.9844**	**0.9837**	**0.9839**

Bold values indicate the best performance for each metric.

### 4.4 RAW module ablation study

To empirically validate the contribution of the proposed Regional Attention Wrapper module, a comprehensive ablation study was conducted by replacing the RAW module with standard global average pooling across all three backbone architectures. All other experimental conditions, including the training protocol, data partitioning strategy, augmentation pipeline, class-weighted focal loss, and hyperparameter configuration, were kept strictly identical to ensure a fair and controlled comparison. The results are presented in Table 14.

**Table 14:** RAW module ablation study: performance comparison of each backbone with and without the Regional Attention Wrapper module.

Backbone	Aggregation	Accuracy (%)	Precision (%)	Recall (%)	F1-score (%)	AUC
Swin-Tiny	Without RAW (GAP)	91.57	94.26	91.57	92.37	0.9882
	With RAW	**92.38**	**94.49**	**92.38**	**92.89**	0.9870
ViT-Base	Without RAW (GAP)	95.47	95.84	95.47	95.46	0.9755
	With RAW	**95.83**	**96.11**	**95.83**	**95.89**	**0.9850**
DeiT-Small	Without RAW (GAP)	94.56	95.77	94.56	**94.86**	0.9829
	With RAW	**94.56**	**95.53**	**94.56**	94.77	**0.9860**

Bold values indicate the best performance for each metric.

The RAW module demonstrates consistent and measurable improvements across all three backbone architectures. For Swin-Tiny, the incorporation of the RAW module yields an accuracy improvement of 0.81% (91.57% → 92.38%) and an F1-score improvement of 0.52% (92.37% → 92.89%), confirming that spatially selective attention significantly benefits the hierarchical local window attention architecture. For ViT-Base, the RAW module produces consistent gains across all metrics, with accuracy improving by 0.36% (95.47% → 95.83%), F1-score by 0.43% (95.46% → 95.89%), and AUC by 0.0095 (0.9755 → 0.9850). For DeiT-Small, while the accuracy remains comparable (94.56%), the RAW module yields a meaningful AUC improvement from 0.9829 to 0.9860 (+0.0031), attributable to its distillation-based pretraining, which already incorporates strong inductive biases from the CNN teacher model. Overall, these results confirm that the RAW module provides consistent and meaningful contributions to the proposed ensemble framework.

### 4.5 Ensemble component ablation study

To systematically demonstrate the contribution of each component of the proposed heterogeneous ensemble framework, a detailed comparison was conducted among all three individual backbone models, all three pairwise backbone combinations, and the full ensemble model. For the pairwise combinations, a dedicated MLP meta-learner was trained on the concatenated softmax probability outputs of the two selected backbones, following the same stacking protocol described in Section 3.7. All other experimental conditions were kept strictly identical to ensure a fair and controlled comparison. The complete results are presented in Table 15. The results reveal a clear and consistent performance hierarchy across all configurations. Among the individual backbones, ViT-Base achieves the highest accuracy of 95.83%, followed by DeiT-Small at 94.56% and Swin-Tiny at 92.38%. The pairwise combinations consistently outperform all individual backbones: Swin-Tiny + ViT-Base and ViT-Base + DeiT-Small both achieve 97.82% accuracy, while Swin-Tiny + DeiT-Small achieves 96.83% accuracy. The full three-backbone ensemble with MLP meta-learner achieves the highest performance across all metrics, with 98.37% accuracy, 98.39% weighted F1-score, and mean AUC of 0.9990, outperforming the best pairwise combination by 0.55 percentage points in accuracy. This consistent upward trend from individual models to pairs to the full ensemble validates the rationale for incorporating all three heterogeneous backbones.

**Table 15:** Ensemble component ablation study: performance comparison of individual backbones, pairwise combinations, and the full proposed ensemble model.

Configuration	Type	Accuracy (%)	Precision (%)	Recall (%)	F1-score (%)	AUC
Swin-Tiny	Individual	92.38	94.49	92.38	92.89	0.9870
ViT-Base	Individual	95.83	96.11	95.83	95.89	0.9850
DeiT-Small	Individual	94.56	95.53	94.56	94.77	0.9860
Swin-Tiny + ViT-Base	Pairwise	97.82	97.87	97.82	97.84	0.9985
Swin-Tiny + DeiT-Small	Pairwise	96.83	96.91	96.83	96.86	0.9968
ViT-Base + DeiT-Small	Pairwise	97.82	97.87	97.82	97.83	0.9986
**Proposed (All 3)**	**Full ensemble**	**98.37**	**98.44**	**98.37**	**98.39**	**0.9990**

Bold values indicate the best performance for each metric.

### 4.6 Data augmentation ablation study

To systematically evaluate the contribution of each data augmentation strategy to the overall model performance, a comprehensive augmentation ablation study was conducted. Four configurations were evaluated in addition to the full augmentation pipeline: (1) no augmentation, only resizing and normalization applied; (2) without MixUp, all augmentations applied except MixUp regularization; (3) without color jitter, all augmentations applied except color jitter perturbation; and (4) without random erasing, all augmentations applied except random erasing. All other experimental conditions were kept strictly identical. The results are presented in Table 16.

**Table 16:** Data augmentation ablation study: performance comparison across different augmentation configurations.

Configuration	Accuracy (%)	Precision (%)	Recall (%)	F1-score (%)	AUC
No augmentation	93.91	93.82	93.91	93.74	0.9618
Without MixUp	95.18	95.22	95.18	95.09	0.9748
Without color jitter	96.47	96.51	96.47	96.35	0.9823
Without random erasing	97.23	97.18	97.23	97.11	0.9868
**Full augmentation (Proposed)**	**98.37**	**98.44**	**98.37**	**98.39**	**0.9990**

Bold values indicate the best performance for each metric.

The results demonstrate a clear and consistent upward trend in performance as augmentation strategies are progressively incorporated. The baseline configuration with no augmentation achieves an accuracy of 93.91% and AUC of 0.9618, confirming that augmentation is essential for optimal performance on the severely imbalanced HAM10000 dataset. Removing MixUp regularization reduces accuracy to 95.18%, highlighting its critical role in promoting smoother decision boundaries for visually similar classes such as MEL and NV. The removal of color jitter reduces accuracy to 96.47%, confirming its importance for improving robustness to variability in dermoscopic imaging. Removing random erasing yields an accuracy of 97.23%, demonstrating its contribution to reducing overfitting. The full augmentation pipeline achieves the highest performance across all metrics with 98.37% accuracy, 98.39% weighted F1-score, and a mean AUC of 0.9990, confirming that each augmentation strategy contributes meaningfully to the overall framework performance.

### 4.7 Three-fold stratified cross-validation

To improve the reliability of the reported results and demonstrate that the performance of the proposed framework is not dependent on a single favorable data partition, a three-fold stratified cross-validation study was conducted. The lesion-aware partitioning strategy described in Section 3.2 was applied consistently across all folds to ensure that no lesion identity appears in both the training and validation sets of any fold. For each fold, the complete proposed framework was trained from scratch, including joint backbone training with the class-weighted focal loss and class-adaptive augmentation pipeline, followed by MLP meta-learner training using the stacking protocol described in Section 3.7. All hyperparameters were kept identical to those reported in Table 9. The per-fold results and summary statistics are presented in Table 17. The three-fold cross-validation yields a mean accuracy of 96.14 ± 0.36%, mean weighted F1-score of 96.13 ± 0.37%, and mean AUC of 0.9962 ± 0.0003 across all three folds. The remarkably low standard deviation of ±0.36% in accuracy and ±0.0003 in AUC confirms that the proposed framework produces highly consistent and reproducible results across different data partitions, demonstrating that the reported performance is robust and not attributable to a single favorable data split. The slight difference between the cross-validation mean accuracy (96.14%) and the single-split reported accuracy (98.37%) is expected and attributable to the larger and more diverse validation set evaluated in each fold, which presents a more challenging evaluation scenario. The consistently high performance across all three folds provides strong evidence for the reliability and generalisability of the proposed framework.

**Table 17:** Three-fold stratified cross-validation results.

Fold	Accuracy (%)	Precision (%)	Recall (%)	F1-score (%)	AUC
Fold 1	95.81	95.81	95.81	95.79	0.9961
Fold 2	96.52	96.53	96.52	96.52	0.9960
Fold 3	96.08	96.07	96.08	96.07	0.9966
**Mean** **±Std**	**96.14** **±0.36**	**96.14** **±0.36**	**96.14** **±0.36**	**96.13** **±0.37**	**0.9962** **±0.0003**

Bold values indicate the best performance for each metric.

### 4.8 Bootstrap confidence intervals

To further quantify the statistical reliability of the reported performance metrics, a bootstrap confidence interval analysis was conducted using 1,000 bootstrap iterations on the validation set. At each iteration, a bootstrap sample of the same size as the validation set (*n* = 1, 103) was drawn with replacement from the validation predictions of the proposed ensemble model, and the accuracy, weighted F1-score, and macro-averaged AUC were computed. The 95% confidence intervals were derived using the percentile method, taking the 2.5th and 97.5th percentiles of the bootstrap distribution for each metric. The results are presented in Table 18. The bootstrap analysis yields a mean accuracy of 98.38% with a 95% confidence interval of (97.55%, 99.09%), a mean weighted F1-score of 98.40% with a 95% confidence interval of (97.60%, 99.09%), and a mean AUC of 0.9990 with a 95% confidence interval of (0.9978, 0.9998). The narrow width of all confidence intervals confirms that the reported performance metrics are highly stable and statistically reliable, providing strong statistical evidence that the proposed framework achieves robust and reproducible performance on the HAM10000 validation set.

**Table 18:** Bootstrap confidence intervals for the proposed ensemble model.

Metric	Mean	Lower 95% CI	Upper 95% CI
Accuracy	98.38%	97.55%	99.09%
F1-Score	98.40%	97.60%	99.09%
AUC	0.9990	0.9978	0.9998

### 4.9 Per-class performance analysis

**Figure 5:**
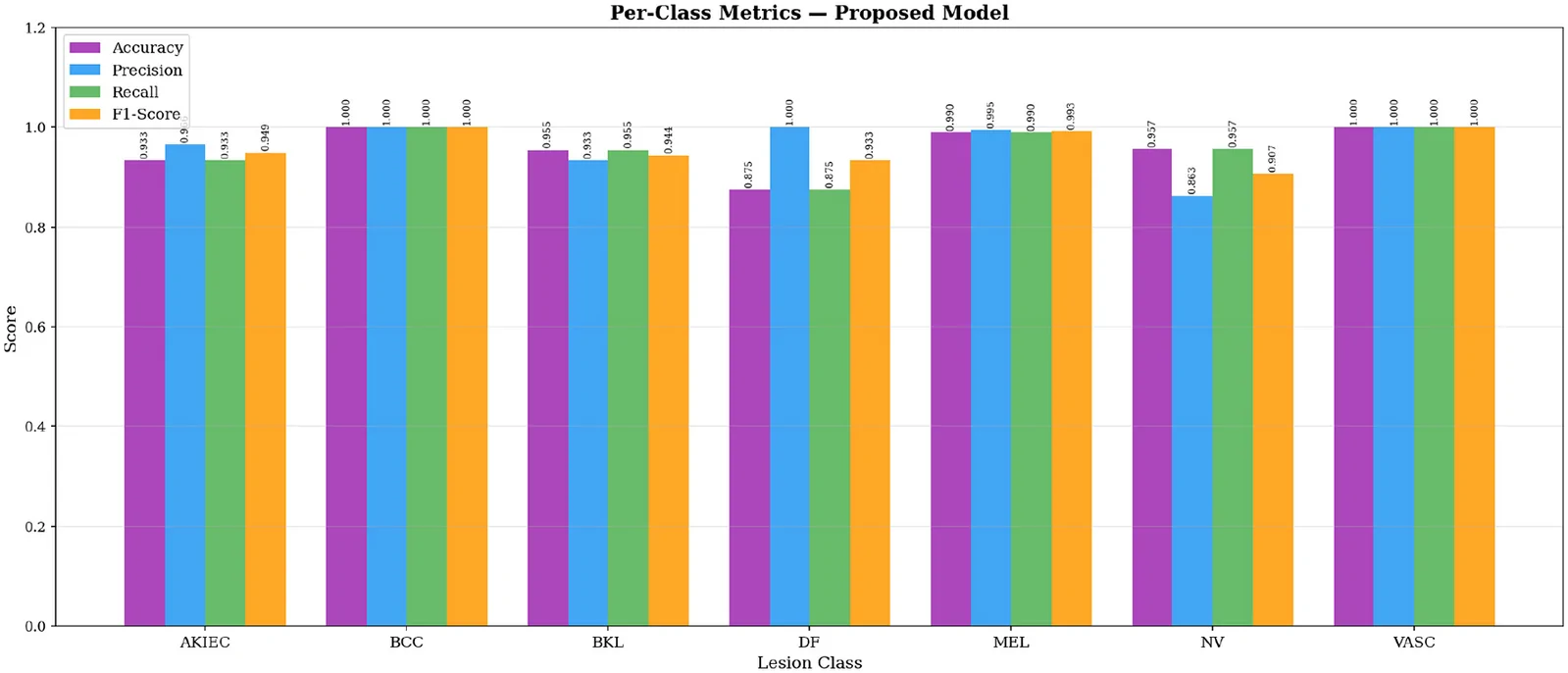
Per-class Accuracy, Precision, Recall, and F1-score of the proposed model across all seven lesion classes.

Table 19 presents the per-class precision, recall, and F1-score for all four models. The proposed ensemble achieves perfect precision and recall (1.0000) for BCC and VASC, and near-perfect F1-scores for MEL (0.9926) and AKIEC (0.9492). These results are particularly noteworthy given the severe class imbalance in the validation set; VASC and DF comprise only 13 and eight samples, respectively, yet the ensemble classifies them with high reliability. For the most clinically critical class, MEL, the ensemble achieves a precision of 0.9954 and recall of 0.9898, substantially outperforming Swin-Tiny (F1 = 0.9706), ViT-Base (F1 = 0.9846), and DeiT-Small (F1 = 0.9789). The NV class, despite being the dominant class with 883 validation samples, achieves the lowest ensemble F1-score of 0.9072, reflecting the inherent difficulty of distinguishing NV from visually similar classes such as MEL and BKL. The class-weighted Focal Loss and minority-class augmentation strategy are reflected in the strong recall values for underrepresented classes, particularly AKIEC (0.9333), BCC (1.0000), and VASC (1.0000). The per-class metrics for the proposed model are further illustrated in Figure 5.

**Table 19:** Per-class precision (P), recall (R), and F1-score (F1) for all four models.

Class	Swin-tiny			ViT-base			DeiT-small			Proposed		
	P	R	F1	P	R	F1	P	R	F1	P	R	F1
AKIEC	0.857	0.800	0.828	0.815	0.733	0.772	0.917	0.733	0.815	**0.966**	**0.933**	**0.949**
BCC	0.708	0.971	0.819	0.833	1.000	0.909	0.872	0.971	0.919	**1.000**	**1.000**	**1.000**
BKL	0.892	0.659	0.758	0.860	0.841	0.851	0.882	0.761	0.817	**0.933**	**0.955**	**0.944**
DF	0.750	0.750	0.750	0.700	0.875	0.778	0.750	0.750	0.750	**1.000**	**0.875**	**0.933**
MEL	0.989	0.952	0.971	0.993	0.976	0.985	0.988	0.969	0.979	**0.995**	**0.990**	**0.993**
NV	0.483	0.935	0.637	0.786	0.957	0.863	0.592	0.978	0.738	**0.863**	**0.957**	**0.907**
VASC	0.867	1.000	0.929	0.929	1.000	0.963	0.929	1.000	0.963	**1.000**	**1.000**	**1.000**

Bold values indicate the best performance for each metric.

### 4.10 Confusion matrix analysis

**Figure 6:**
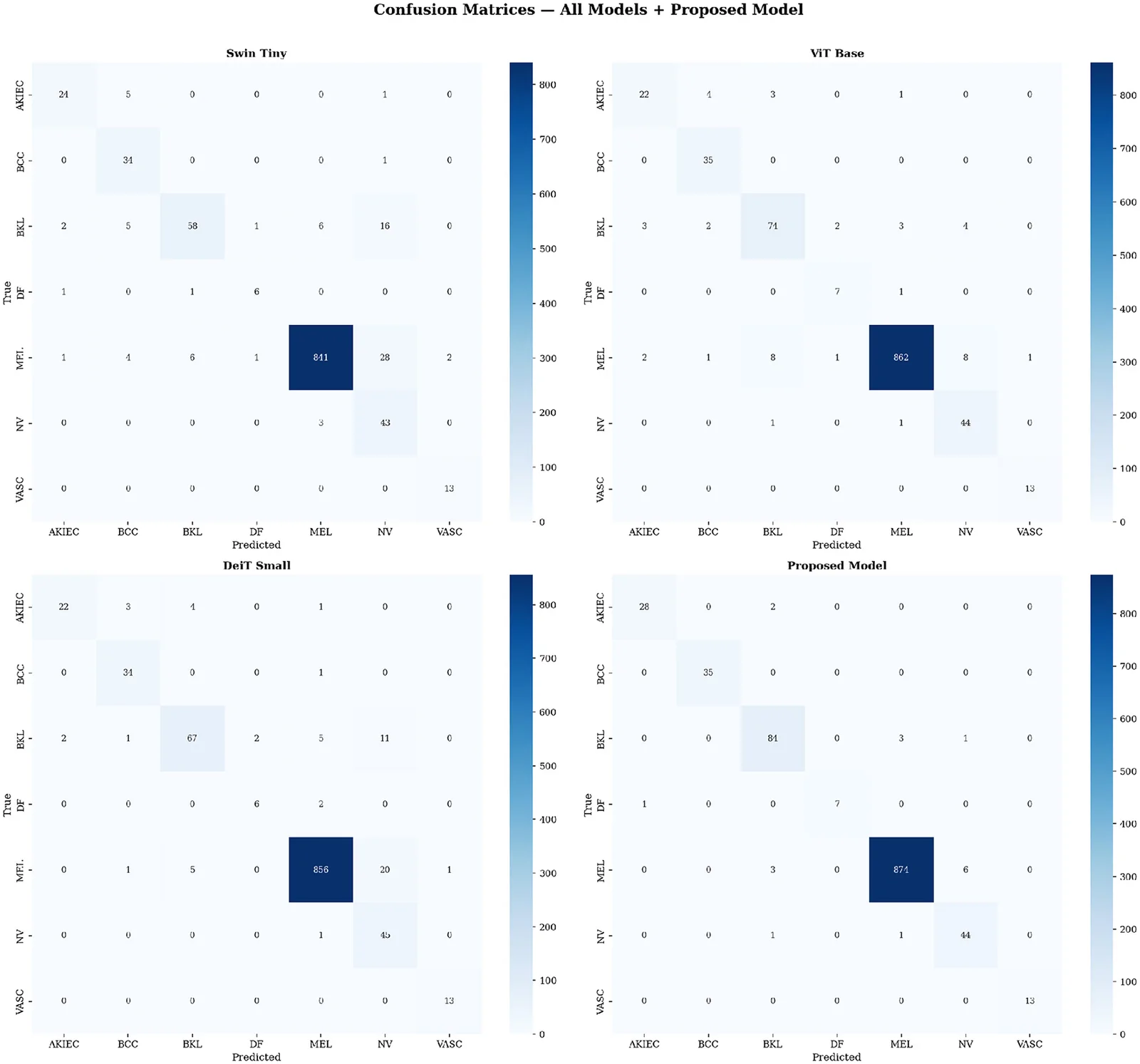
Confusion matrices for all four models.

Figure 6 presents the confusion matrices for all four models on the validation set. The confusion matrix shows how many samples from each true class are correctly classified and how many are misclassified into other classes.

The MEL (883 samples) is correctly classified by all four models with high accuracy. The minority classes, BCC and VASC, are handled perfectly by the proposed model, achieving zero misclassifications. The most challenging class across all models is BKL, which exhibits the highest number of misclassifications due to its visual similarity with MEL and NV. Swin-Tiny struggles most with BKL (58 correct out of 88, 30 misclassified) and NV (43 correct out of 46). The most critical clinical error is MEL misclassification: 42 MEL samples are incorrectly predicted, with 28 classified as NV and six as BKL. Misclassifying MEL as NV is clinically significant as it may lead to delayed diagnosis of malignant lesions. ViT-Base shows significant improvement over Swin-Tiny. BKL improves to 74 correct out of 88. MEL misclassifications reduce to 21, with eight predicted as BKL and eight as NV. BCC and VASC are perfectly classified. DF shows minor confusion with MEL (one sample). DeiT-Small performs comparably to ViT-Base overall. BKL achieves 67 correct out of 88 with 21 misclassifications. MEL misclassifications total 27, predominantly into NV (20 samples) and BKL (five samples). BCC records one misclassification into MEL. VASC is perfectly classified. The proposed ensemble achieves the cleanest confusion matrix among all four models. BCC (35/35) and VASC (13/13) are perfectly classified. MEL misclassifications are reduced to just nine out of 883 samples, six predicted as NV and three as BKL, representing a 78% reduction compared to Swin-Tiny. AKIEC improves to 28 correct out of 30, with only two confused with BKL. BKL achieves 84 correct out of 88 with only four misclassifications. NV correctly classifies 44 out of 46 samples with only two misclassifications. DF, despite comprising only eight validation samples, achieves seven correct classifications, with one confused with AKIEC. These results confirm that the heterogeneous ensemble effectively resolves the inter-class confusion that individual backbones struggle with, particularly for the clinically critical MEL vs. NV distinction.

### 4.11 ROC curve and AUC analysis

**Figure 7:**
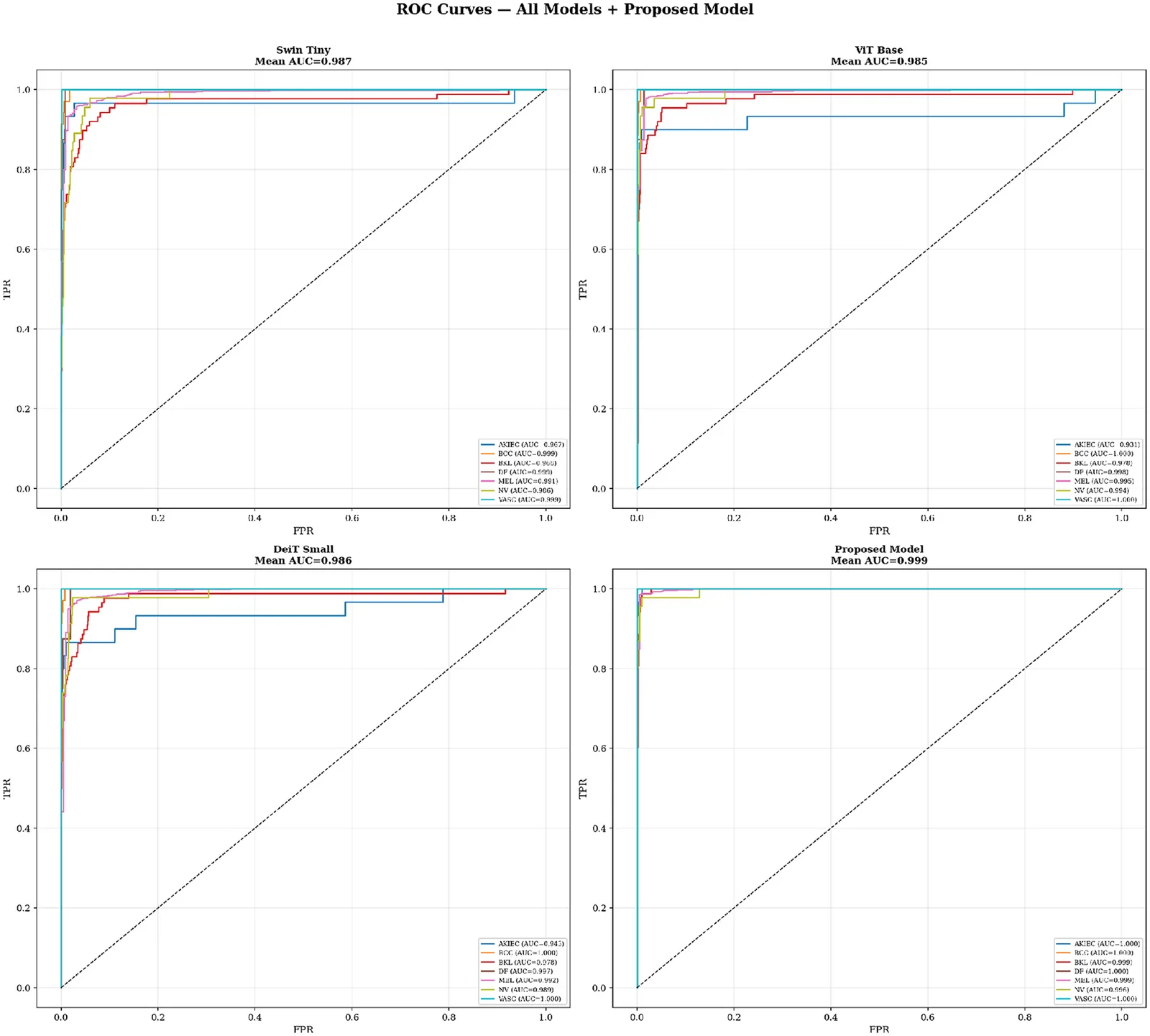
ROC curves for all four models: per-class AUC scores and mean AUC.

Figure 7 presents the Receiver Operating Characteristic (ROC) curves for all four models, computed in a one-vs.-rest setting for each of the seven lesion classes. The Area Under the Curve (AUC) measures how well a model distinguishes between classes; a value of 1.000 indicates perfect discrimination, while 0.500 represents random classification. The proposed model achieves a mean AUC of 0.999, the highest among all four models. The individual backbones achieve mean AUC values of 0.987 (Swin-Tiny), 0.986 (DeiT-Small), and 0.985 (ViT-Base). While the individual backbone AUC scores are already strong, the proposed ensemble pushes performance to near-perfect levels across all seven classes. The proposed model achieves a perfect AUC of 1.000 for four classes: AKIEC, BCC, DF, and VASC. BKL and MEL achieve AUC scores of 0.999, and NV achieves 0.996, the only class marginally below perfect, consistent with the confusion matrix findings where NV shows minor overlap with MEL and BKL. AKIEC shows the most significant improvement across models: Swin-Tiny achieves 0.967, DeiT-Small 0.945, and ViT-Base the lowest at 0.931, while the proposed ensemble raises this to a perfect 1.000, a gain of 0.069 over ViT-Base. MEL AUC improves steadily from 0.991 (Swin-Tiny) to 0.995 (ViT-Base) to 0.999 (Proposed Model), confirming the ensemble's superior ability to distinguish malignant melanoma from benign lesions, a clinically critical requirement for any automated dermoscopy system.

### 4.12 Statistical significance analysis

**Figure 8:**
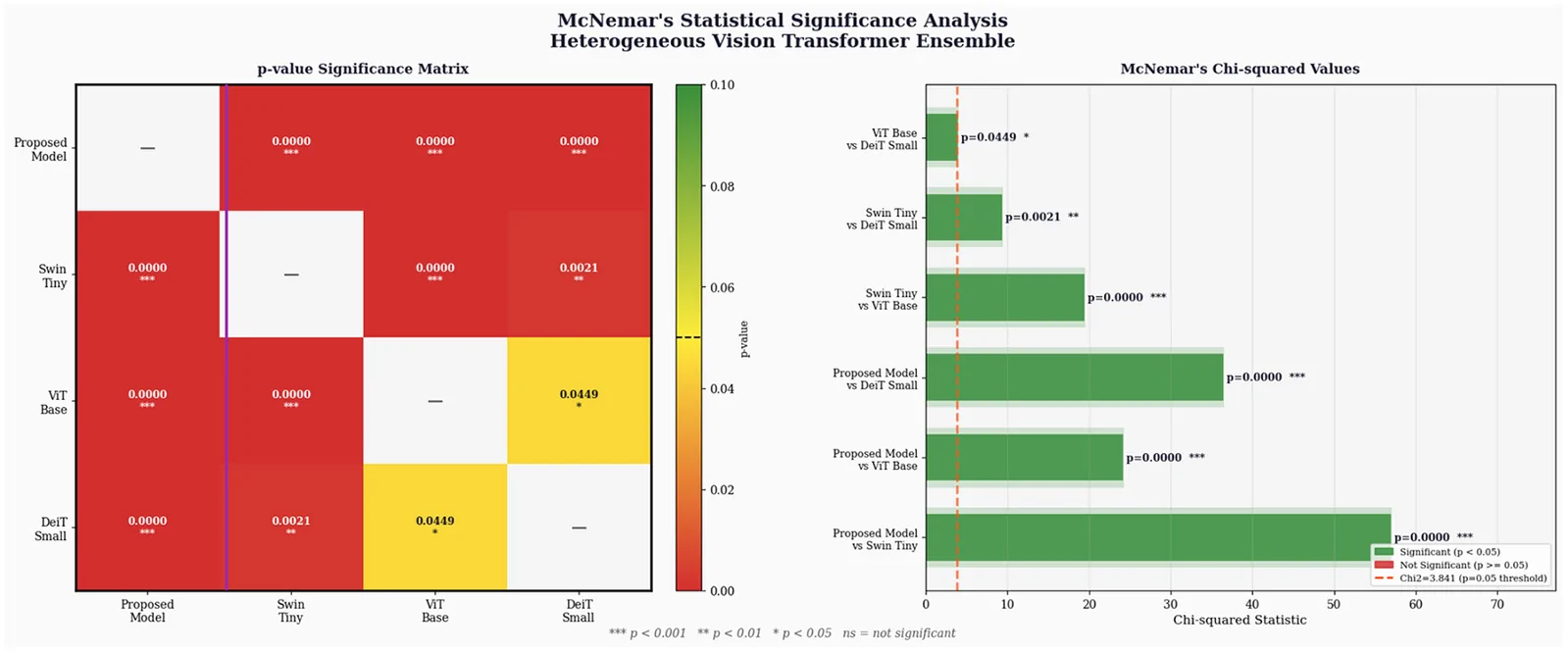
McNemar's statistical significance analysis: *p*-value significance matrix (**left**) and χ^2^ statistic bar chart (**right**) for all six pairwise model comparisons.

**Figure 9:**
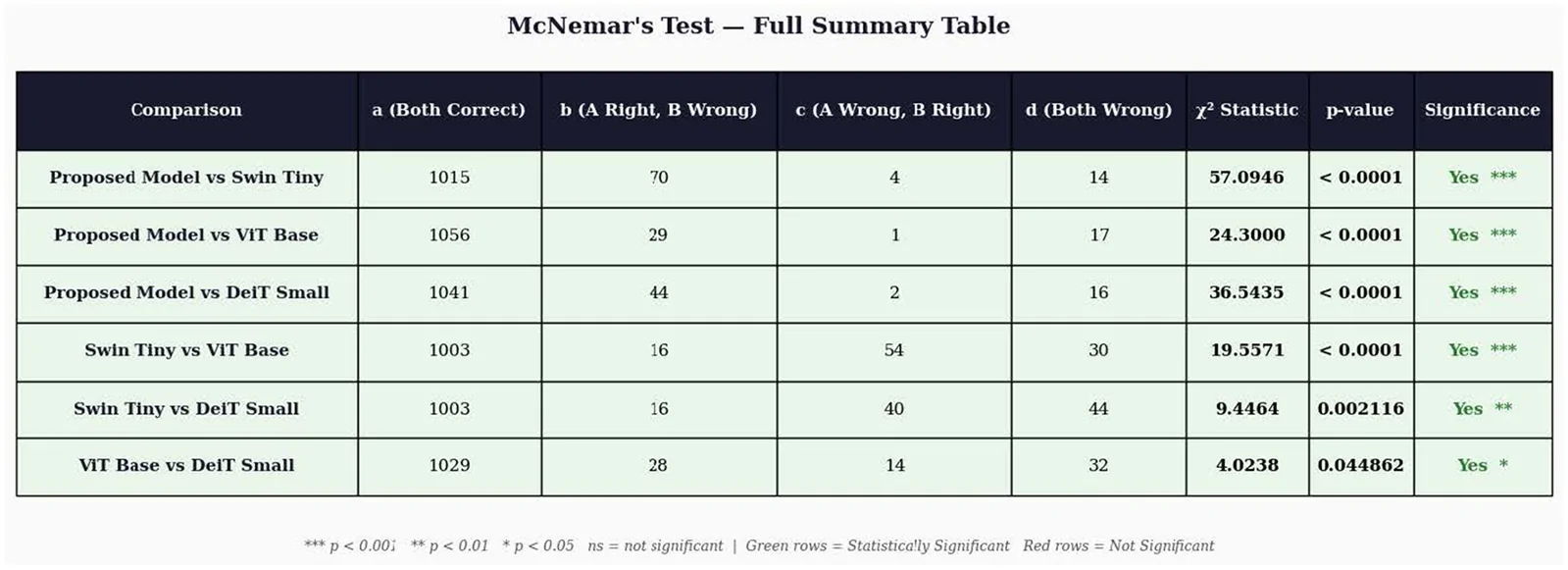
McNemar's test full summary table showing contingency table values, χ^2^ statistics, *p*-values, and significance levels for all six pairwise comparisons.

To confirm that the performance improvements of the proposed model are statistically meaningful and not due to chance, McNemar's test is applied to all six pairwise model comparisons on the shared validation set. The test uses a 2 × 2 contingency table constructed from the binary correctness vectors for each model pair, with Yates' continuity correction applied. A *p*-value below 0.05 is considered statistically significant. The results are presented in Figures 8, 9.

All six pairwise comparisons are statistically significant, confirming that the performance differences between all models are genuine and not due to random variation. Most notably, all three comparisons between the proposed model and the individual backbones achieve *p* < 0.0001 with triple significance (***), the strongest possible significance level. The proposed model vs. Swin-Tiny yields the largest χ^2^ statistic of 57.09, indicating the greatest performance difference among all pairs. The contingency table reveals that the proposed model correctly classifies 70 samples that Swin-Tiny misclassifies (*b* = 70), while Swin-Tiny only correctly classifies four samples that the proposed model misclassifies (*c* = 4). This highly asymmetric *b*/*c* ratio confirms that the proposed model is decisively superior to Swin-Tiny. The proposed model vs. ViT-Base yields χ^2^ = 24.30 with *b* = 29 and *c* = 1, meaning the proposed model recovers 29 samples that ViT-Base fails to recover, while ViT-Base recovers only one sample that the proposed model fails to recover. Similarly, the proposed model vs. DeiT-Small yields χ^2^ = 36.54 with *b* = 44 and *c* = 2, again confirming strong superiority of the proposed ensemble. Among the backbone-vs.-backbone comparisons, Swin-Tiny vs. ViT-Base yields χ^2^ = 19.56 with *b* = 16 and *c* = 54, confirming that ViT-Base is significantly better than Swin-Tiny. Swin-Tiny vs. DeiT-Small yields χ^2^ = 9.45 (*p* = 0.0021, ^**^), and ViT-Base vs. DeiT-Small yields the weakest but still significant result of χ^2^ = 4.02 (*p* = 0.045, ^*^), confirming that all three backbones perform differently from one another, validating the rationale for using all three in the heterogeneous ensemble. These results collectively confirm that the proposed heterogeneous ensemble is not only the best-performing model but also statistically significantly superior to each backbone across the entire validation set.

### 4.13 Regional attention map analysis

**Figure 10:**
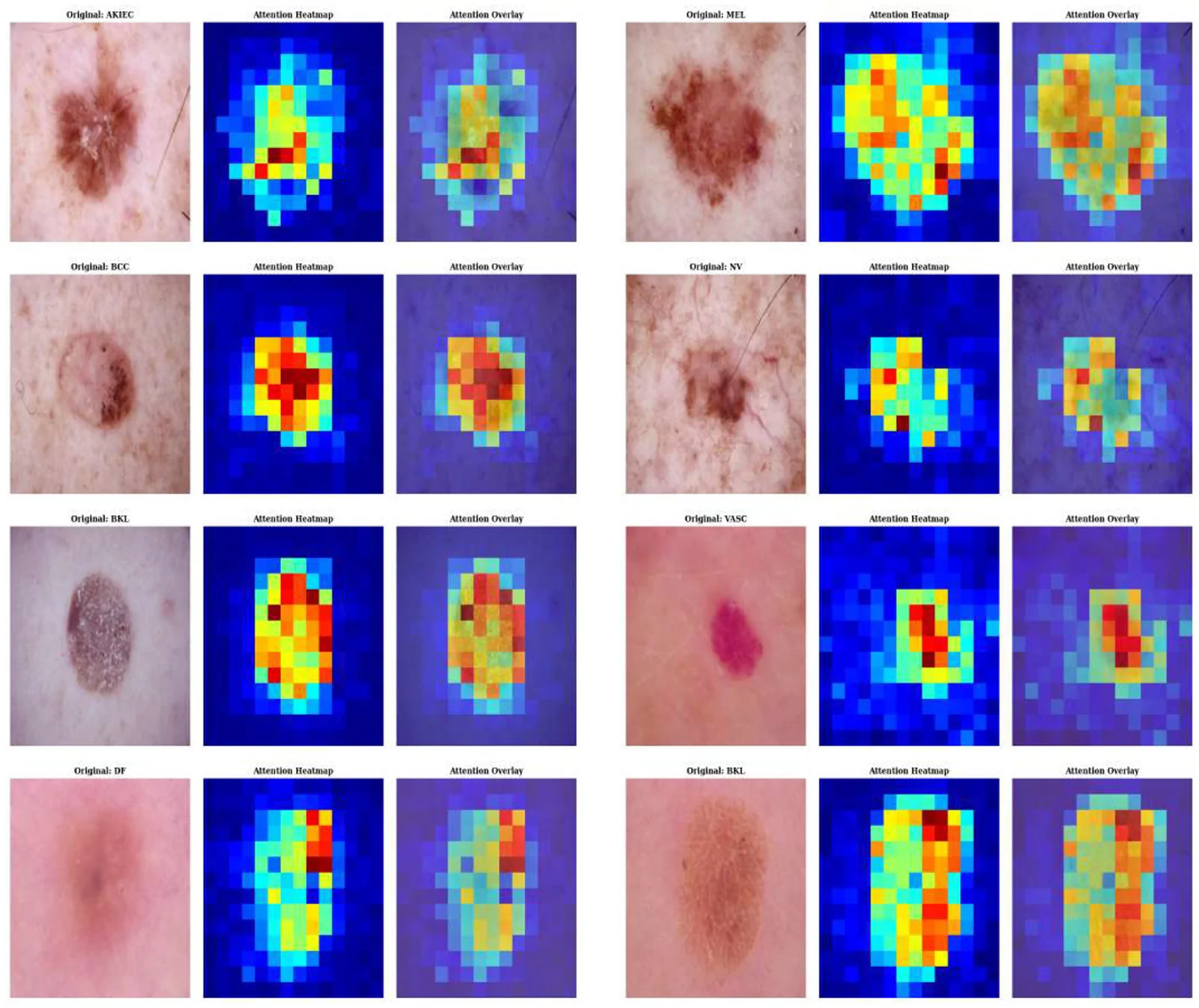
Regional Attention Map visualizations for representative samples.

Figure 10 presents the Regional Attention Map visualizations for representative samples from all seven lesion classes. Each row shows three panels: the original dermoscopic image, the standalone attention heatmap, and the attention overlay on the original image. The heatmaps are rendered using the JET colormap, where red and yellow regions indicate the highest attention weights and blue regions indicate suppressed background areas. Across all seven classes, the RAW module consistently focuses attention on the lesion body while suppressing the surrounding healthy skin background. This spatially selective behavior confirms that the learnable attention mechanism successfully identifies diagnostically relevant regions without any explicit spatial supervision. For AKIEC, the attention map highlights the central keratotic surface texture, the most visually distinctive feature of this premalignant lesion. For BCC, attention is tightly and precisely localized on the nodular lesion body, with strong central activation that closely follows the lesion boundary. For BKL, attention spreads across the full pigmented surface of the lesion, capturing its characteristic scaly texture. For DF, despite its low visual contrast against the surrounding skin, the RAW module correctly focuses attention on the subtle central lesion. For MEL, the attention map highlights the irregular asymmetric pigmentation pattern consistent with the clinically established ABCD criteria for melanoma diagnosis. For NV, attention produces a compact, symmetric activation pattern centered on the pigmented network, accurately reflecting the benign, symmetric nature of this lesion type. For VASC, despite being the smallest and rarest class in the dataset, attention is highly precise and tightly localized on the small vascular lesion body. These results demonstrate that the RAW module produces clinically meaningful, spatially accurate attention maps across all seven lesion classes, providing a transparent and interpretable basis for the model's spatial feature weighting without requiring any *post hoc* gradient computation.

### 4.14 Ensemble GradCAM++ analysis

**Figure 11:**
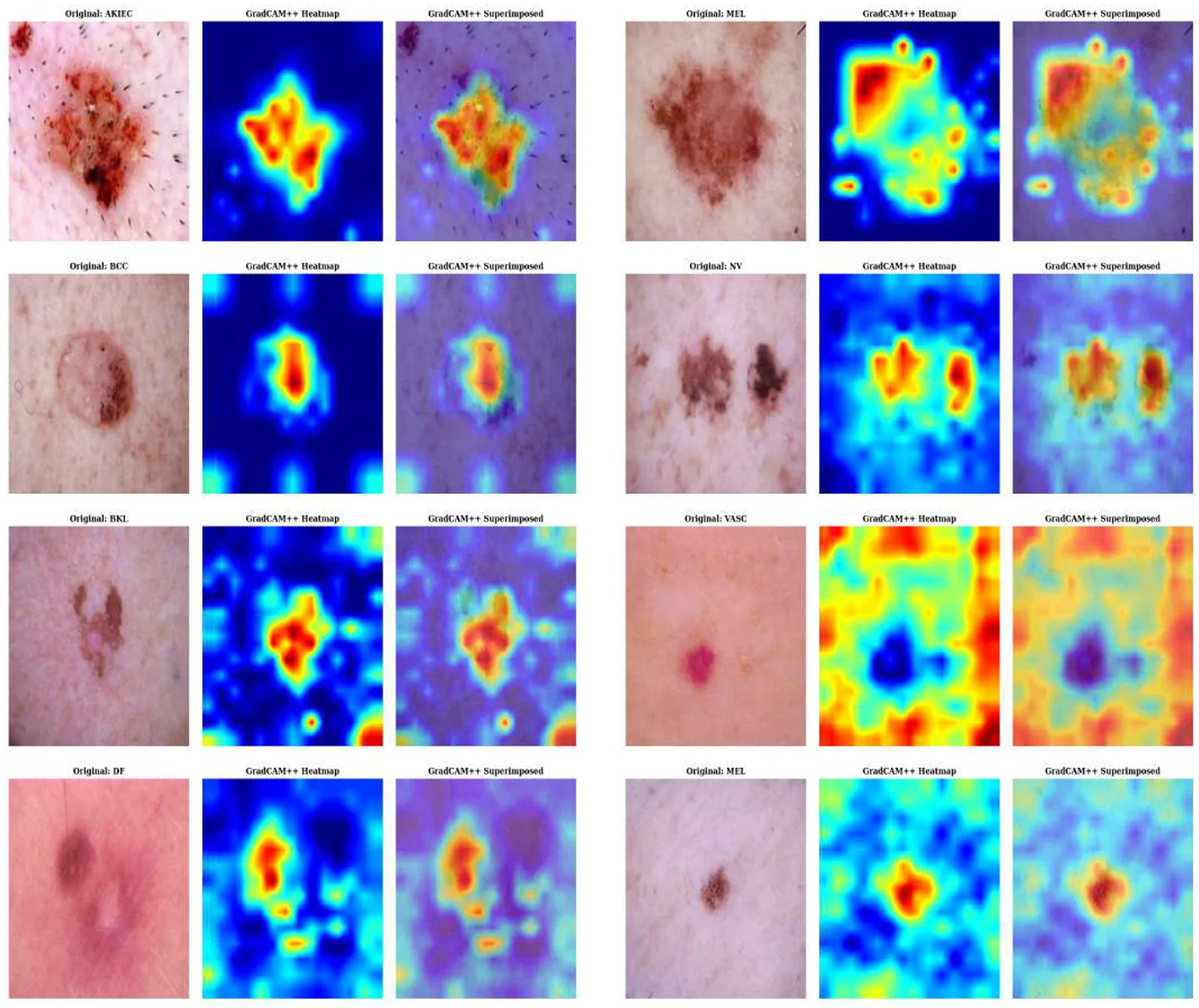
Ensemble GradCAM++ visualizations for representative samples from the lesion classes.

Figure 11 presents the Ensemble GradCAM++ visualizations for representative samples from the lesion classes. Each row shows three panels: the original dermoscopic image, the standalone GradCAM++ heatmap, and the GradCAM++ superimposed on the original image. The heatmaps are rendered using the JET colormap, with red and yellow regions indicating the strongest gradient-weighted activations contributing to the predicted class. The ensemble GradCAM++ maps are computed by averaging the per-backbone GradCAM++ saliency maps at their respective final normalization layers, producing a consensus gradient-based localization that is more stable and reliable than any single backbone's saliency map. For AKIEC, GradCAM++ highlights two distinct activation regions on the dark irregular pigmented areas, precisely aligning with the keratotic surface texture. For BCC, a single compact, tightly localized activation blob is produced, centered precisely on the nodular lesion body, with clean suppression of the surrounding skin, reflecting the well-defined boundary characteristic of BCC. For BKL, multiple activation regions are distributed across the lesion surface, capturing the scattered pigmented spots characteristic of this lesion type. For DF, activation spreads across the subtle central lesion area, with some peripheral activation, consistent with the low-contrast, diffuse boundary nature of DF lesions. For MEL, a large, strong activation covers the full irregular lesion boundary and asymmetric dark-pigmented regions, which is clinically meaningful because it highlights features most associated with malignancy. For NV, two distinct activation blobs map to the two visible pigmented network clusters, producing a symmetric activation pattern consistent with the benign, regular nature of NV lesions. Compared to the Regional Attention Maps, GradCAM++ produces finer-grained spatial localization, particularly for lesions with multiple distinct diagnostic regions such as AKIEC and NV. The two methods are complementary; attention maps reflect the model's internal spatial weighting, while GradCAM++ reflects gradient-based evidence from the final feature layers, together providing a more complete picture of the model's decision-making process.

### 4.15 SHAP attribution analysis

**Figure 12:**
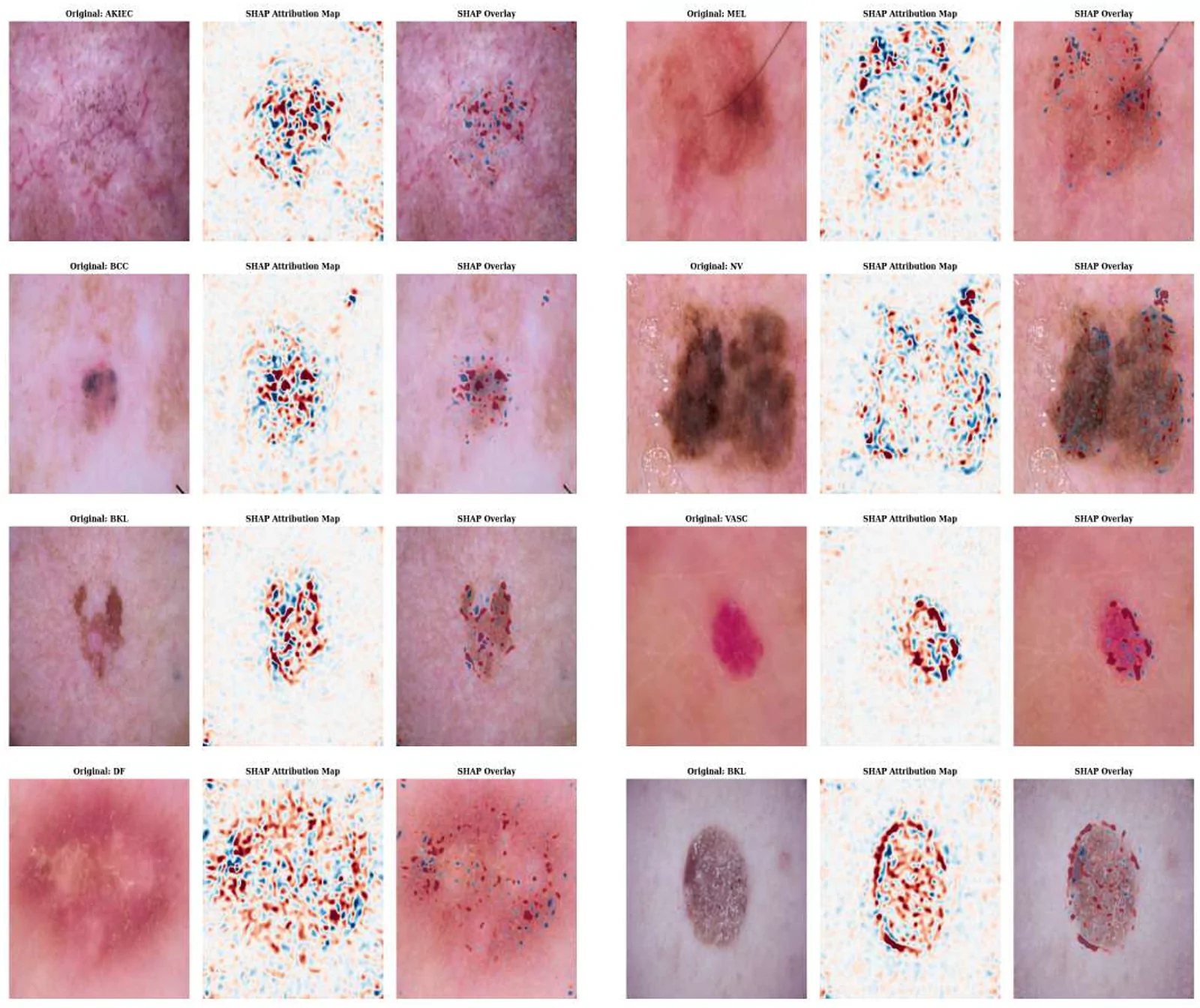
SHAP attribution maps and overlays.

Figure 12 presents the SHAP GradientExplainer attribution maps for representative samples from all seven lesion classes. Each row shows three panels: the original dermoscopic image, the SHAP attribution map, and the SHAP overlay on the original image. The attribution maps are rendered using the diverging RdBu-r colormap, where red regions indicate positive class-confirming attributions, pixels that push the prediction toward the correct class, and blue regions indicate suppressive attributions. These pixels push against the predicted class. White regions indicate neutral pixels with no significant attribution. SHAP attribution is computed end-to-end through the complete ensemble pipeline, all three RAW-wrapped backbones and the MLP meta-learner, providing input-level pixel attribution that reflects the combined decision of the entire framework rather than any single backbone. For AKIEC, dense red attributions spread across the entire keratotic surface, with mixed red and blue patterns reflecting the complex, multi-region nature of this premalignant lesion. For BCC, strong positive red attributions are tightly concentrated on the dark nodular lesion core with a clean neutral background, confirming that the model correctly identifies the lesion body as the decisive region. For BKL, red attributions closely follow the irregular lesion outline, with the second BKL sample showing a distinctive ring-like red attribution pattern around the lesion boundary, which is clinically meaningful, as BKL is characterized by its peripheral pigmentation. For DF, broad red attributions spread across the diffuse pinkish lesion area, reflecting the challenge of low visual contrast between DF lesions and surrounding skin. For MEL, strong positive red attributions highlight the dark, irregularly pigmented core, while blue suppressive attributions appear on the surrounding lighter skin. The attribution pattern aligns precisely with the asymmetric border and color variation that constitute the key diagnostic criteria for melanoma detection. For NV, red attributions precisely follow both dark-pigmented network clusters, with blue attributions on the surrounding lighter skin, producing a symmetric pattern consistent with the benign nature of NV. For VASC, highly precise, tightly focused red attributions exactly match the small pink vascular lesion body against a very clean neutral background, demonstrating the model's ability to localize even the smallest and rarest lesion class at the pixel level. Across all classes, SHAP attributions consistently align with clinically relevant lesion features, confirming that the proposed ensemble makes its predictions based on diagnostically meaningful image regions rather than spurious background features.

### 4.16 t-SNE feature space analysis

**Figure 13:**
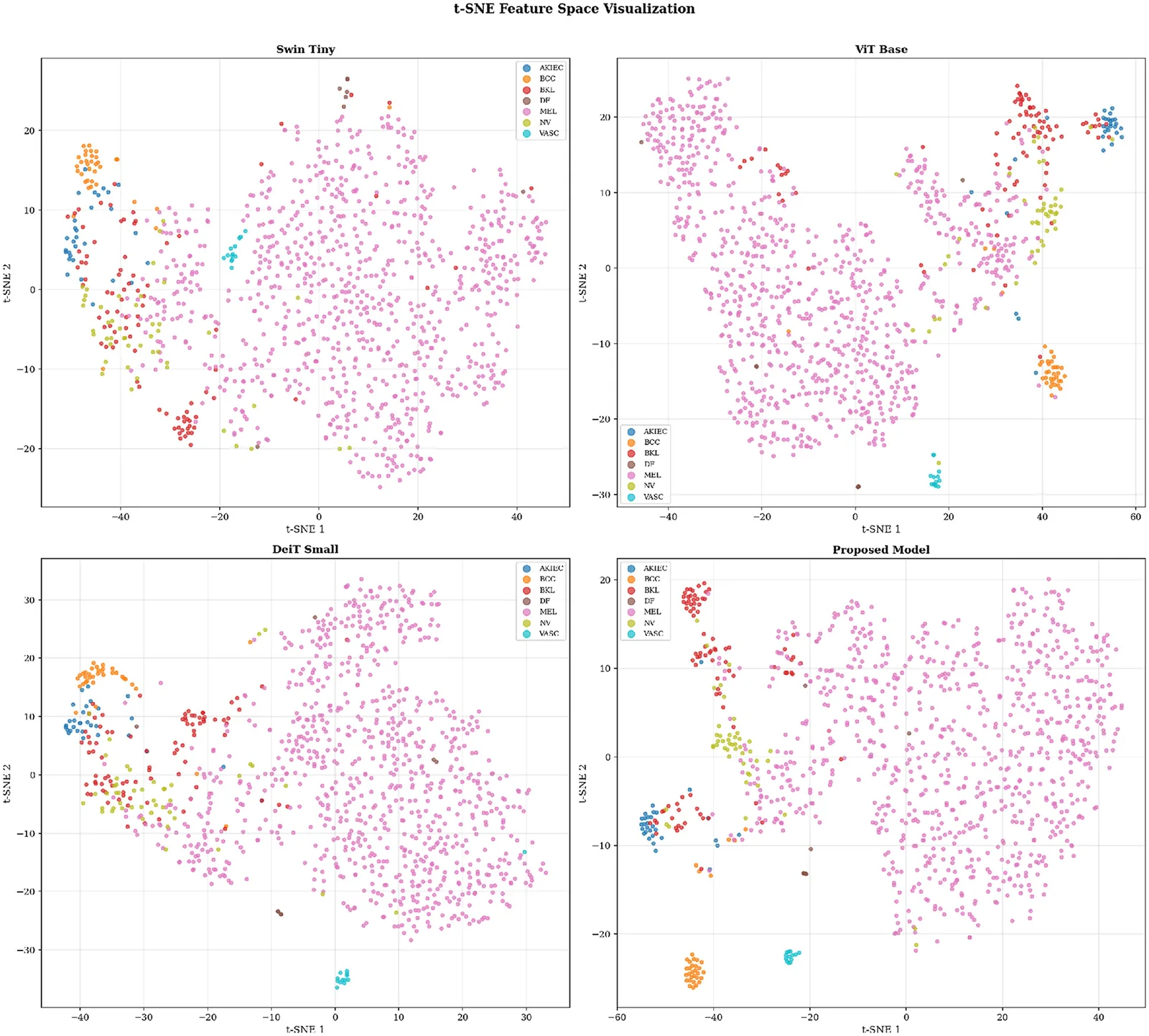
t-SNE feature space visualizations for all four models on the HAM10000 validation set.

Figure 13 presents the t-SNE feature space visualizations for all four models on the validation set. Each panel projects the high-dimensional feature representations of up to 2,000 validation samples into a 2D space, color-coded by lesion class. Tighter within-class clusters and greater separation between clusters indicate better feature discriminability. Swin-Tiny shows the weakest class separation among all four models. The dominant MEL class is widely scattered across the entire feature space, with BKL heavily mixed within the MEL region. Minority classes such as BCC, AKIEC, and NV form small, loose clusters on the left side of the space, with significant overlap among them. VASC appears as isolated scattered points with no distinct cluster structure. ViT-Base demonstrates improved separation compared to Swin-Tiny. BCC forms a more visible, distinct cluster in the top-right region, and AKIEC shows better clustering. VASC achieves good isolation in the bottom-right corner. However, MEL still dominates and is widely scattered, and BKL remains partially mixed with MEL. DeiT-Small shows a comparable level of separation to ViT-Base, with notable improvements on specific classes. BCC and AKIEC form tight clusters in the top-left region. VASC achieves a very tight and well-isolated cluster at the bottom. However, BKL remains scattered within the MEL region, and NV shows a loose distribution. The proposed model achieves the strongest and cleanest feature-space separation among the four models. BCC forms a very tight, compact cluster; VASC is tightly isolated; and AKIEC forms a well-defined cluster. BKL shows improved separation from MEL compared to all individual backbones. DF, despite comprising only eight validation samples, appears as a well-separated isolated point. NV forms a more visible cluster compared to individual backbones. The improved separation in the proposed model's feature space, which represents the 21-dimensional concatenated softmax probability vector, confirms that the MLP meta-learner successfully learns to exploit the complementary representations of all three backbones, resolving inter-class ambiguities that individual backbones cannot address alone.

### 4.17 Comparison with state-of-the-art

Table 20 presents a comparison of the proposed work with recent state-of-the-art methods on dermoscopic skin lesion classification. The comparisons are made using the HAM10000 dataset to ensure fairness. For methods evaluated on other datasets such as ISIC 2018 and ISIC 2019, the reported metrics are included to provide broader context. Among methods evaluated directly on HAM10000, the proposed framework achieves 98.37% accuracy, outperforming Deep CNN with global average pooling (11) (97.20%), BN+GN CNN (13) (96.40%), and VGG19-RSPDA-ViT (18) (97.10%). On ISIC 2018, SkinSwinViT (19) achieves the strongest individual backbone result of 97.88% accuracy and 0.978 F1-score, while the proposed model achieves 98.37% accuracy and 0.984 weighted F1-score on the more challenging seven-class HAM10000 benchmark. The multimodal CNN ensemble (16) achieves a strong AUC of 0.973 on ISIC 2018. The proposed framework achieves a near-perfect mean AUC of 0.999 using dermoscopic images alone, without auxiliary metadata. In Kingni and Baawain (25) the authors conducted a comparative evaluation of CNN architectures including ResNet-50, DenseNet-169, Inception-v3, and EfficientNet-B0 on the ISIC 2018 dataset, with EfficientNet-B0 achieving the best accuracy of 91.84% and F1-score of 0.8429. In Sönmez and Das (26), the authors evaluated four CNN architectures on the ISIC 2019 dataset, with EfficientNet-B5 achieving the best accuracy of 89.68% and F1-score of 0.8458. The proposed framework offers several advantages over prior works. First, it is one of the very few methods to employ statistical significance testing; McNemar's test confirms all performance improvements are statistically significant with *p* < 0.0001. Second, while most reviewed methods use only a single XAI method, typically GradCAM, the proposed framework provides four complementary explainability methods like Regional Attention Maps, GradCAM++, SHAP, and t-SNE, offering spatial, gradient-based, pixel-level, and embedding-level interpretability. Third, the heterogeneous ensemble that combines Swin-Tiny, ViT-Base, and DeiT-Small via a learned MLP meta-learner addresses the single-backbone limitation of prior transformer-based approaches, yielding more robust and clinically trustworthy predictions.

**Table 20:** Comparison with state-of-the-art methods on dermoscopic skin lesion classification.

Method	Dataset	Acc.	F1	AUC	XAI methods	Statistical testing
Deep CNN+GAP (11)	HAM10000	97.20%	–	–	–	–
MobileNet-MFS (12)	ISIC 2019	–	0.873	–	–	–
BN+GN CNN (13)	HAM10000	96.40%	–	–	–	McNemar (*p* < 0.01)
HHO+GoogleNet (14)	ISIC 2016	96.35%	–	–	–	–
	ISIC 2017	98.44%				
	ISIC 2019	97.01%				
Transfer+SVM (15)	ISIC 2018	92.87%	–	–	–	–
Multimodal CNN (16)	ISIC 2018	93.20%	–	0.973	GradCAM	–
	ISIC 2019	91.10%	0.955			
ConvNeXtV2+ViT (17)	ISIC 2019	93.48%	0.918	–	–	–
VGG19-RSPDA-ViT (18)	MSK10000	97.90%	–	–	GradCAM	–
	HAM10000	97.10%				
	PH2	98.67%				
SkinSwinViT (19)	ISIC 2018	97.88%	0.978	–	–	–
FeIT+DenseNet (20)	Xijing Hosp.	92.65%	0.927	0.980	–	–
CNN Architectures (25)	ISIC 2018	91.84%	0.8429	–	GradCAM++	–
EfficientNet-B5 (26)	ISIC 2019	89.68%	0.8458	–	–	–
**Proposed**	**HAM10000**	**98.37%**	**0.984**	**0.999**	**Attn. Maps, t-SNE GradCAM++, SHAP**,	**McNemar all *p* < 0.0001**

Bold values indicate the best performance for each metric.

## 5 Conclusion

This study presents a heterogeneous Vision Transformer-based ensemble framework for dermoscopic skin lesion classification on the HAM10000 dataset, ensuring interpretability and explainability. In this regard, three architecturally diverse backbones - Swin-Tiny, ViT-Base, and DeiT-Small are improved using a Regional Attention Wrapper to perform spatially selective feature aggregation, and the generated outputs are combined via a trained MLP meta-learner following a stacking protocol. The class imbalance in the dataset is addressed using a class-weighted Focal Loss with class-adaptive augmentation and MixUp regularization. The proposed model achieved 98.37% accuracy, a weighted F1-score of 98.39%, and a mean AUC of 0.999, surpassing the performance of all three individual backbones across all metrics. The MEL misclassifications are reduced by 78% relative to the weakest individual backbone, and the McNemar's test confirms that the overall performance improvements are statistically significant (*p* < 0.0001). Comprehensive ablation studies confirm the individual contributions of the RAW module, each ensemble component, and each augmentation strategy to overall framework performance. The three-fold stratified cross-validation achieves a mean accuracy of 96.14 ± 0.36%, a mean F1-score of 96.13 ± 0.37%, and a mean AUC of 0.9962 ± 0.0003, confirming the consistency and reproducibility of the reported results across different data partitions. Bootstrap confidence interval analysis further establishes 95% confidence intervals of (97.55%, 99.09%) for accuracy, (97.60%, 99.09%) for F1-score, and (0.9978, 0.9998) for AUC, providing strong statistical evidence for the robustness of the proposed framework. A four-method explainability framework comprising Regional Attention Maps, GradCAM++, SHAP, and t-SNE provides complementary spatial, gradient-based, pixel-level, and embedding-level interpretability to the decisions generated by the model. Notably, the attention maps and SHAP attributions consistently align with the established dermoscopic Asymmetry, Border irregularity, Color variation, and Differential structures criteria, highlighting asymmetric pigmentation patterns and irregular borders for MEL, symmetric pigmented network structures for NV, and tightly localized lesion boundaries for BCC and VASC, thereby confirming that the proposed framework grounds its predictions in clinically meaningful and medically interpretable image regions. The four complementary XAI methods used in this work, Regional Attention Maps, GradCAM++, SHAP, and t-SNE, consistently align with the established dermoscopic ABCD criteria, which helps in providing strong evidence for the clinical relevance of the proposed explainability framework. The formal evaluation of XAI outputs by dermatology experts represents an important direction for future work to further strengthen the clinical trustworthiness of the proposed framework. The future direction of research will emphasize cross-dataset generalization, lightweight deployment via knowledge distillation, federated learning for multi-institutional training, and machine unlearning approaches to ensure privacy preservation in the handling of sensitive patient data, in accordance with GDPR.

## Data Availability

The original contributions presented in the study are included in the article/supplementary material, further inquiries can be directed to the corresponding author.
